# Proteomic and transcriptomic analyses identify apo-transcobalamin-II as a biomarker of overall survival in osteosarcoma

**DOI:** 10.3389/fonc.2024.1417459

**Published:** 2024-10-18

**Authors:** Ryan A. Lacinski, Sebastian A. Dziadowicz, Clark A. Roth, Li Ma, Vincent K. Melemai, Brody Fitzpatrick, Edwin Chaharbakhshi, Tanya Heim, Ines Lohse, Karen E. Schoedel, Gangqing Hu, Nicolas J. Llosa, Kurt R. Weiss, Brock A. Lindsey

**Affiliations:** ^1^ Department of Orthopaedics, West Virginia University School of Medicine, Morgantown, WV, United States; ^2^ West Virginia University Cancer Institute, West Virginia University School of Medicine, Morgantown, WV, United States; ^3^ Department of Microbiology, Immunology, and Cell Biology, West Virginia University School of Medicine, Morgantown, WV, United States; ^4^ Bioinformatics Core, West Virginia University School of Medicine, Morgantown, WV, United States; ^5^ Department of Orthopaedic Surgery, University of Pittsburgh, Pittsburgh, PA, United States; ^6^ Department of Pathology, University of Pittsburgh Medical Center, Pittsburgh, PA, United States; ^7^ Department of Orthopaedic Surgery, Johns Hopkins University School of Medicine, Baltimore, MD, United States

**Keywords:** osteosarcoma, plasma, proteomics, transcobalamin-II, immunotherapy

## Abstract

**Background:**

The large-scale proteomic platform known as the SomaScan® assay is capable of simultaneously measuring thousands of proteins in patient specimens through next-generation aptamer-based multiplexed technology. While previous studies have utilized patient peripheral blood to suggest serum biomarkers of prognostic or diagnostic value in osteosarcoma (OSA), the most common primary pediatric bone cancer, they have ultimately been limited in the robustness of their analyses. We propose utilizing this aptamer-based technology to describe the systemic proteomic milieu in patients diagnosed with this disease.

**Methods:**

To determine novel biomarkers associated with overall survival in OSA, we deployed the SomaLogic SomaScan® 7k assay to investigate the plasma proteomic profile of naive primary, recurrent, and metastatic OSA patients. Following identification of differentially expressed proteins (DEPs) between 2-year deceased and survivor cohorts, publicly available databases including Survival Genie, TIGER, and KM Plotter Immunotherapy, among others, were utilized to investigate the significance of our proteomic findings.

**Results:**

Apo-transcobalamin-II (APO-TCN2) was identified as the most DEP between 2-year deceased and survivor cohorts (Log2 fold change = 6.8, P-value = 0.0017). Survival analysis using the Survival Genie web-based platform indicated that increased intratumoral *TCN2* expression was associated with better overall survival in both OSA (TARGET-OS) and sarcoma (TCGA-SARC) datasets. Cell-cell communication analysis using the TIGER database suggested that *TCN2*+ Myeloid cells likely interact with marginal zone and immunoglobin-producing B lymphocytes expressing the TCN2 receptor (CD320) to promote their proliferation and survival in both non-small cell lung cancer and melanoma tumors. Analysis of publicly available OSA scRNA-sequencing datasets identified similar populations in naive primary tumors. Furthermore, circulating APO-TCN2 levels in OSA were then associated with a plasma proteomic profile likely necessary for robust B lymphocyte proliferation, infiltration, and formation of intratumoral tertiary lymphoid structures for improved anti-tumor immunity.

**Conclusions:**

Overall, APO-TCN2, a circulatory protein previously described in various lymphoproliferative disorders, was associated with 2-year survival status in patients diagnosed with OSA. The relevance of this protein and apparent immunological function (anti-tumor B lymphocyte responses) was suggested using publicly available solid tumor RNA-sequencing datasets. Further studies characterizing the biological function of APO-TCN2 and its relevance in these diseases is warranted.

## 1 Introduction

The current mainstay for clinical management of solid tumors, including the determination of cancer type and staging, is predicated on the analysis of tumor specimens collected through invasive biopsy and/or surgical resection (1). Recent characterization of these specimens through next-generation genomic sequencing technologies has not only helped guide the selection of targeted therapeutics in the age of personalized medicine and precision oncology (2–4), but has also been a major driver of cancer biomarker discovery (5, 6). The collection of tumor specimens for these analyses, however, can often pose critical challenges including obtaining sufficient sample quantity (biopsy), adequately characterizing tumors which have metastasized to multiple, often unresectable locations, and repeatedly monitoring therapeutic response over time (1).

Conversely, liquid biopsies, including the collection of patient blood, saliva, or urine, are diagnostic modalities offering inherent advantages over surgically collected tumor specimens. These advantages include their minimal invasiveness in addition to the possibility of serial sampling longitudinally throughout the course of disease (1, 7). The most comprehensively evaluated liquid biopsy to date is patient peripheral blood and its liquid (plasma, serum) and cellular components. Numerous biomarkers isolated from blood specimens including circulating tumor cells (CTCs) (8, 9), tumor exosomes (10, 11), and circulating tumor DNA (ctDNA) (12, 13) have offered invaluable insight into disease processes, prognosis, and therapeutic response for a variety of solid tumors (14, 15), including pediatric malignancies such as sarcoma (16–18).

Large-scale proteomic technologies (19), capable of simultaneously characterizing hundreds to thousands of proteins in these specimens, have also gained significant traction in the field of cancer biomarker research (20). While numerous techniques have been established (21, 22), next-generation aptamer-based multiplexed proteomic technology, first published by Gold et al. in 2010, has shown extraordinary promise. These aptamer-based platforms alleviate some of the inherent limitations of previous proteomic techniques such as mass spectrometry (MS) by offering increased sample throughput, larger dynamic ranges of detection, and lower average coefficients of variation, all while necessitating minimal sample volumes (23). The basis of this technology lies in the development of chemically modified aptamers, which form complex three-dimensional matrices with robust specificity to their target proteins. This new class of aptamers, known as Slow Off-rate Modified Aptamers (SOMAmers), are an evolution of the previously described short single-stranded oligonucleotides identified by Systemic Evolution of Ligands by Exponential (SELEX) enrichment in the early 1990s (23–25).

SOMAmers and the SomaScan® platform (26, 27), developed commercially by SomaLogic, Inc. (Boulder, CO, USA), have now been used to characterize a variety of cancers, including but not limited to hepatocellular carcinoma (28), colorectal cancer (29), pancreatic cancer (30), glioma (31), lung cancer (32), oral squamous cell carcinoma (33), and ovarian cancer (34). The platform has expanded its characterization from approximately 800 proteins in 2010 to well over 7000 proteins today, offering extensive insight into nearly all biological pathways relevant to human disease (35). Due to the SomaScan’s wide ranging assessment of proteins involved in the immune system including cytokine signaling, signaling by interleukins, and immunoregulatory pathways, among others, the platform has even been used to identify circulating proteins associated with immunotherapy response in diseases such as melanoma (36). As immunotherapies begin to dominate the world of oncology, systemic assessments of the immune system, in real time, are likely critical to monitor immunotherapeutic response and better predict clinical success. To our knowledge, no study has yet utilized the SomaScan® platform for proteomic biodiscovery in osteosarcoma (OSA).

OSA is the most common primary pediatric bone malignancy (37, 38). Patients with localized disease, treated with surgical resection and neoadjuvant/adjuvant multi-drug chemotherapy (39), display five-year survival rates greater than 75% (40, 41). Unfortunately, this rate decreases to approximately 25% in those with advanced disease, most commonly in the form of metastases to the lung (42). Numerous studies have been conducted to determine biomarkers of prognostic or diagnostic value in OSA using liquid biopsies (43–47). Of utmost relevance, serum biomarkers (48) including tumor necrosis factor (TNF) (49) and other interleukins (50), vascular endothelial growth factor (VEGF) (51–53) and angiogenin (ANG) (54), macrophage migration inhibitory factor (MIF) and T-Cell Immunoglobulin and Mucin Domain-Containing Protein 3 (TIM-3) (55), as well as various chemokines including C-X-C motif chemokine ligand (CXCL)4, CXCL6, and CXCL12 (56) have shown preliminary promise as diagnostic and/or prognostic biomarkers in this disease. Additionally, analysis of plasma proteomic profiles using surface-enhanced laser desorption/ionization-time of flight (SELDI-TOF) MS identified two proteins (amyloid protein A and transthyretin) involved in the innate immune system associated with positive response to chemotherapy (57). While limited in the robustness of their analyses, these studies ultimately support that characterization of the OSA systemic proteome can offer unique insight into disease progression, therapeutic response, and prognosis in this disease.

**Figure 1:**
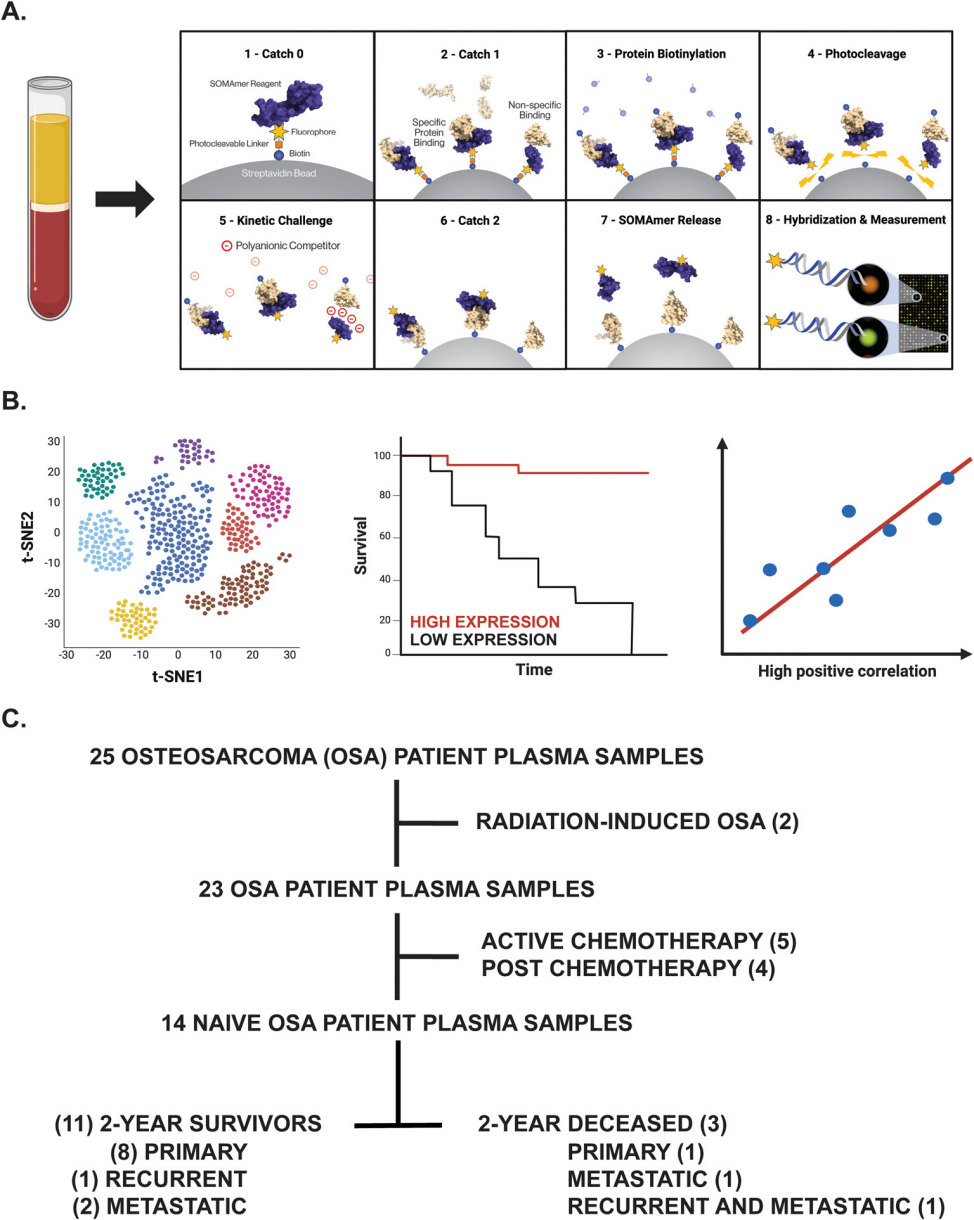
Overview of experimental design. **(A)** Proteomic investigation of OSA patient plasma samples using the SomaLogic SomaScan® 7k assay. Step-by-step process by which the SomaScan platform measures circulatory proteins (provided by SomaLogic Inc.). **(B)** Downstream analyses including cell identification using scRNA-seq analysis, overall survival analysis in publicly available datasets, and correlation analyses to support identified protein’s role or association in solid tumors. Figure generated using BioRender.com. **(C)** Tree diagram depicting those OSA patient plasma samples selected for downstream analyses in this study.

To determine novel biomarkers associated with overall survival in OSA, we utilized the SomaScan® 7k assay to investigate the plasma proteomic profile of primary, recurrent, and metastatic OSA patients. Plasma samples, isolated from patients with confirmatory diagnosis of OSA and treated at the University of Pittsburgh, Department of Orthopaedic Surgery, were processed and analyzed for the simultaneous quantification of over 7000 circulatory proteins (**Figure 1A**). Following the identification of differentially expressed proteins (DEPs) between 2-year deceased and survivor patient cohorts, various analyses were then conducted to suggest their biological relevance in OSA including investigation of publicly available bulk and single cell (sc)RNA-seq datasets, further associations with overall and progression free survival in solid tumors, as well as correlations with previously published biomarkers and gene signatures (**Figure 1B**).

## 2 Materials and methods

### 2.1 Patients, sample collection, and processing

The human subject research protocol and subject informed consent were reviewed and approved by the University of Pittsburgh Medical Center Institutional Review Board (IRB STUDY 19060152). According to IRB guidelines, informed consent was obtained from all participants (**Table 1**) before study entry. Peripheral blood specimens were collected from primary, recurrent, and metastatic OSA patients diagnosed from 2014-2021 via venipuncture into Cell-Free DNA BCT tubes (Cat. #218996, Streck, La Vista, NE, USA). Blood components (buffy coat, plasma) were then separated for storage following centrifugation, with time from sample collection to processing averaging 17.4 ± 3.6 hours (ranging 0.04 to 44.06 hours).

**Table 1:** Patient information for the 14 naive OSA patients analyzed within our proteomic biodiscovery analysis.

Patient	Sex	Vital Status	Age at sample collection	Treatment Status	Disease Status (complex)	Disease Status (simple)	2-year survival	Spin down time (hrs)
1	F	AWD	13.00	Naive	Primary	Primary	Yes	26.59
2	F	DWD	14.00	Naive	Metastatic	Advanced	No	27.16
3	M	AWD	14.00	Naive	Metastatic	Advanced	Yes	27.04
4	M	AWD	15.00	Naive	Primary	Primary	Yes	22.85
5	F	AWD	16.00	Naive	Primary	Primary	Yes	6.16
6	M	DWD	19.00	Naive	Recurrent	Advanced	Yes	0.04
7	F	DWD	19.00	Naive	Recurrent, metastatic	Advanced	No	3.93
8	M	AWD	20.00	Naive	Primary	Primary	Yes	30.52
9	F	AWD	21.00	Naive	Primary	Primary	Yes	4.87
10	M	AWD	22.00	Naive	Primary	Primary	Yes	8.27
11	M	AWD	32.00	Naive	Primary	Primary	Yes	28.85
12	F	AWD	33.00	Naive	Primary	Primary	Yes	6.78
13	M	DWD	47.00	Naive	Primary	Primary	No	44.06
14	M	DWD	69.00	Naive	Metastatic	Advanced	Yes	6.28

F - female, M - male, AWD - alive with disease, DWD - deceased with disease.

### 2.2 SomaLogic SomaScan® 7k assay

Profiling of the OSA proteome was performed using the SomaScan® 7k assay, a highly multiplexed aptamer-based proteomic technology capable of determining over 7,000 protein measurements, at SomaLogic headquarters according to a previous publication (58). Briefly, frozen OSA Streck plasma samples were thawed and then buffer exchanged twice into SomaLogic Assay Buffer using Zeba 7kDa MWCO spin desalting columns (Cat. #89882, Thermo Scientific, Waltham, MA, USA) according to manufacturer’s instructions. These buffer-exchanged plasma samples (diluted to 20%, 0.5%, and 0.005%) were then placed in a 96-well plate with a mix of thousands of chemically modified aptamers called SOMAmer® Reagents. These SOMAmers®, consisting of a 5’ fluorophore, photocleavable linker, and biotin on streptavidin-coated beads, form complex three-dimensional shapes which bind to epitopes on their target protein with high affinity and specificity. Following the formation of SOMAmer-protein complexes, unbound proteins are washed away, captured proteins are labeled with biotin, and the SOMAmer-protein complexes are released by photocleavage with ultraviolet light in a buffer containing an unlabeled polyanionic competitor. The biotin-labeled SOMAmer-protein complexes are captured on a second set of streptavidin-coated beads before releasing SOMAmer reagents in a denaturing buffer. The amount of available protein epitope is read by hybridizing the SOMAmer reagents in the SomaScan Assay eluate to complementary sequences on a DNA microarray. Here, fluorescence is measured as relative fluorescence units (RFUs) and reflects overall epitope abundance (**Figure 1A**). The RFU readout is normalized by three non-consecutive steps including hybridization control normalization, intraplate median signal normalization, and median signal normalization to a reference and then delivered in a tab-delimited text file (.adat extension) for analysis using SomaLogic’s web-based tool known as DataDelve Statistics alongside the DataDelve Stats v1.0 User Guide. A T-test was used to compare the means of two groups (such as 2-year deceased and survivor cohorts). Considering the exploratory nature of our study and limited OSA patient samples analyzed, differentially expressed proteins (DEPs) between comparative groups were identified as having a Log2FC > 0.585 or < -0.585 and P-value < 0.05. Median normal patient plasma RFU measurements (provided by SomaLogic) for both APO- and HOLO-transcobalamin-II (TCN2) are graphed on the associated box plot and are representative of a healthy, control patient population derived from previous publication (58). Considering these median values were determined using normal patient ethylenediaminetetraacetic acid (EDTA)-plasma samples that had been centrifuged and stored 2-10 hours post-blood collection (as opposed to the Streck-collected specimens analyzed here), they should only serve as a general guideline to the major findings from our analyses. A Volcano plot depicting those DEPs between comparative groups was generated using GraphPad Prism 10 (GraphPad Software, Boston, MA, USA).

### 2.3 Reactome pathway analysis

A pathway analysis of those DEPs between 2-year deceased and survivor cohorts (all OSA and advanced OSA cohorts) was conducted using the Reactome Pathway Analysis Tool (https://reactome.org/PathwayBrowser/, version 87) according to the Reactome User Guide (Docs – Analysis Tools) and previous publications (59–61). Briefly, all proteins with Log2FC > 0.585 or < -0.585 and P-value < 0.05 were inputted into the identifier list alongside their associated Log2FC. For consistency, proteins identified by the same SOMAmer, each with a unique sequencing ID, however, sometimes detecting multiple proteins [example seq.3714-49 quantifying both Creatine kinase M-type and Creatine kinase B-type heterodimer (P12277|P06732 or CKB|CKM)], were inputted in the identifier list multiple times with the same corresponding Log2FC. Following data input and use of default analysis settings (projection to human without interactors), the pathway analysis results (.csv) and analysis report (pdf) were exported. A genome-wide overview plot is used to highlight the Reactome pathways over- (blue) or underrepresented (yellow) in the input dataset, with light grey signifying non-significant pathways. Significant Reactome pathways for the given data input were identified as having an FDR < 0.05.

### 2.4 Survival Genie overall survival and CIBERSORTx TIL correlation analysis

The Survival Genie web-interface (https://bhasinlab.bmi.emory.edu/SurvivalGenie/) was used for both overall survival and CIBERSORTx tumor-infiltrating lymphocyte (TIL) analyses according to the user guide and a previous publication (62). Briefly, single gene analyses [transcobalamin-II (*TCN2*), alpha 2-HS glycoprotein (*AHSG*)] were conducted using the National Cancer Institute (NCI) Therapeutically Applicable Research to Generate Effective Treatments-Osteosarcoma (TARGET-OS) and The Cancer Genome Atlas (TCGA)-Sarcoma (SARC) databases. Here, single gene normalized expression [fragments per kilobase of transcript per million mapped reads (FPKM)] from primary tumors is associated with overall survival through Cutp estimated martingale residuals (63) and patient stratification into low and high expressing groups using the survMisc package (64). Resulting Kaplan-Meier (KM) survival curves with high (red) and low (blue) group stratification are compared using the log-rank test, with log-rank P-value < 0.05 considered statistically significant (64). The provided Forest plot details the association between the high and low groups [stratified by cut point (CP)] based on the Cox Proportional Hazards regression model (survMisc package). Hazard ratio (HR) with 95% confidence interval as well as the associated Wald-test (HR) and log-rank (LR) P-values are reported (64). The relative fraction of TILs was estimated using the tumor-infiltrating immune cell type matrix LM22 gene signature from bulk tumor FPKM gene expression data and the CIBERSORTx deconvolution method (65). A Pearson correlation matrix of deconvoluted immune cell RNA-seq gene expression data and our genes of interest are reported. Shape (square or circle) denotes significance while color denotes positive (red) or negative (blue) correlation with the genes of interest (*TCN2*, *AHSG*).

### 2.5 TIGER scRNA-seq analysis

The Tumor Immunotherapy Gene Expression Resource (TIGER) portal (http://tiger.canceromics.org/#/) was used to investigate the target genes of interest [*TCN2*, transcobalamin receptor (*CD320*)] according to the user guide and a previous publication (66). Briefly, after inputting the genes of interest, the “Single-cell Immunity” tab was used to investigate gene expression across various scRNA-seq datasets of the TIGER database. Here, the Cell Type Marker (Log2FC) diagram represents differential expression of various clusters and subclusters in each scRNA-seq dataset of the TIGER database. These cells, organized by cancer type, dataset ID, main lineage, cell type, and Log2FC, were then sorted for highest Log2FC expression of the target genes. The top 20 clusters with increased gene expression were reported. After identifying the various cell types (myeloid, plasma, and B cells) and cancers [non-small cell lung cancer (NSCLC) – dataset NSCLC (67), NSCLC1 (68), NSCLC5 (69), NSCLC6 (70)], skin cutaneous melanoma (SKCM) – dataset SKCM1 (71)] with increased expression of the target gene(s), a subsequent cell-cell communication analysis was performed. Briefly, cell-cell communication in the NSCLC (67) and SKCM1 (71) datasets was investigated due to expression of both genes of interest (*TCN2*, *CD320*) across different cell types. Following navigation to the “Single-cell Immunity” tab and selection of the NSCLC dataset, a cell-cell communication analysis was conducted for the Mye_C1_CCL18 cluster (with greatest expression of *TCN2*) and the B_C10_MZB1 cluster (with greatest expression of *CD320*). The resulting heatmap displays the expression of all receptor-ligand interactions between any cell type of interest and its location [example – tumor, peritumoral (if applicable)]. The top three cell-cell interactions with the greatest total effect score are reported. Upon toggling the effect score between cell types of interest (example Mye_C1_CCL18 | B_C10_MZB1), the gene expression of the corresponding receptor-ligand interactions between those cells is displayed and sorted from greatest to least expression. For the top three cell-cell interactions, the top 20 receptor-ligand gene interactions (present intratumorally) and their expression were reported. A similar analysis was conducted for both the Mye_C4_C1QA (with greatest expression of *TCN2*) and the B_C2_IGHG1 (with greatest expression of *CD320*) of the SKCM1 dataset without responder/non-responder stratification (71).

### 2.6 scRNA-seq analysis of OSA tumor specimens

Raw OSA scRNA-seq data of GSE162454 (naive primary tumors) and GSE152048 (chemotherapy treated primary, recurrent, and metastatic tumors) were downloaded from the Gene Expression Omnibus (GEO) under their accession number. The FASTQ files were mapped to the GRCh38/hg38 reference genome using Cell Ranger (10X Genomics, Pleasanton, CA, USA, version 6.1.2). Individual cloupe files for each patient sample were then analyzed using the Loupe Browser (10X Genomics, version 7.0.1) according to the 10X Genomics Support guidelines. Briefly, each cloupe file was reanalyzed to filter barcodes through thresholds by Unique Molecular Identifiers (UMIs), threshold by Features, and mitochondrial UMIs. Considering the sheer heterogeneity of the processed samples, this filtering was performed on a sample-to-sample basis. On average, for the GSE162454 dataset, each sample was filtered by UMIs with a min = 666.67 ± 105.41 and max = 74166.67 ± 16654.16, threshold by Features with a min = 500.00 ± 0.00 and max = 9500.00 ± 500.00, and mitochondrial UMIs (%) with a min = 0.00 ± 0.00% and max = 31.33 ± 0.88%. This filtering resulted in approximately 10.3 ± 1.1% of barcodes removed from each sample (range 7.4-14.7%), in accordance with guidelines. On average, for the GSE152048 dataset, each sample was filtered by UMIs with a min = 502.18 ± 1.00 and max = 35000.00 ± 5680.91, threshold by Features with a min = 500.00 ± 0.00 and max = 5681.82 ± 527.74, and mitochondrial UMIs (%) with a min = 0.00 ± 0.00% and max = 20.91 ± 1.63%. This filtering resulted in approximately 13.0 ± 1.5% of barcodes removed from each sample (range 4.6-20.7%), again, in accordance with guidelines. Following barcode filtering, clusters were identified in each sample of both the GSE162454 and GSE152048 datasets according to canonical marker expression of the major cell types identified by Liu et al. in a previous publication including Myeloid cells 1/2 (*LYZ*, *CD68*), NK/T cells (*CD2*, *CD3D*, *CD3E*, *CD3G*, *GNLY*, *NKG7*, *KLRD1*, *KLRB1*), CAFS (*COL1A1*, *ACTA2*, *VIM*), Plasmocytes (*IGHG1*, *MZB1*), Osteoblastic OSA cells (*ALPL*, *RUNX2*, *IBSP*), Osteoclasts (*ACP5*, *CTSK*), Endothelial cells (*EGFL7*, *PLVAP*), and B cells (*MS4A1*, *CD79A*) (72). The expression of *TCN2* and *CD320* was then assessed across all major cellular clusters via visual inspection of t-distributed Stochastic Neighbor Embedding (t-SNE) plots before further downstream analysis. The expression of *TCN2* and *CD320* on Myeloid cells and Plasmocytes/B cells clusters, respectively, was then investigated. For quantification of *TCN2*+ Myeloid cells and *CD320*+ Plasmocytes and B cells, the “Feature Min” function of the Loupe Browser (displaying the minimum expression value of the features in each cluster’s associated feature set when all features are expressed) was utilized. Here, for consistency across patient samples, only those cells expressing all features within a designated feature set (example: *TCN2*+ Myeloid cells with feature set *LYZ*, *CD68*, and *TCN2*) were quantified. Representative t-SNE plots with log normalized expression of the gene or feature gene set of interest are presented. In addition to total number of cells, the percent (%) of total cells and percent (%) of *TCN2*+ Myeloid cells (relative to all Myeloid cells) were calculated by dividing the cell population of interest by total number of filtered cells (barcodes) or by total number of Myeloid cells, respectively. Bar graphs quantifying abundance of various cell types across OSA specimens analyzed were generated using GraphPad Prism 10.

### 2.7 SomaLogic proteomic correlation analyses

Various correlations between plasma APO-TCN2 and previously described soluble markers of plasma cell maintenance (73) and B cell activation (74) [Interleukin (IL)-6, IL-6 receptor(R)a, IL-6Rb, CXCL12, TNF, IL2RA, TNF Superfamily Member 13b (TNFSF13B) or B-cell activating factor (BAFF), TNF Receptor Superfamily Member 13B (TNFRSF13B) or Transmembrane Activator and CAML Interactor (TACI), TNF Receptor Superfamily Member 17 (TNFRSF17) or B-cell maturation antigen (BCMA)], proteins critical for TLS formation [lymphotoxin alpha 1 beta 2 (LTa1b2), Fc Receptor Like 5 (FCRL5), Selectin L (SELL), TNF Superfamily Member 14(TNFSF14)] (75), circulating immunoglobulin (Ig) levels (IgA, IgD, IgE, IgM, J chain, and IgG), as well as proteins of the 12-chemokine (12-CK) tertiary lymphoid structure (TLS) signature (75) [exception – CCL4 (not measured)] were conducted. Briefly, using GraphPad Prism 10, scatter plots representing all naive OSA patient samples (n = 14) with APO-TCN2 (RFU) on the x-axis and values of various other circulatory proteins (RFU) on the y-axis were constructed. A Spearman correlation analysis was conducted using GraphPad Prism 10 with the correlation coefficient (R) and associated P-value (P) presented on each scatter plot.

### 2.8 KM Plotter Immunotherapy survival and correlation analysis

Using the Kaplan-Meier (KM) Plotter Immunotherapy online database (https://kmplot.com/analysis/), both *TCN2* and *CD320* were evaluated for overall and progression free survival in solid tumors (esophageal, gastric, head and neck, melanoma, lung, and urothelial cancer) treated with various immunotherapies (76). Specimens from all solid tumors, collected pre-treatment, irrespective of immunotherapy target [anti-programmed death cell protein 1 (PD-1), anti-programmed death ligand 1 (PD-L1), and anti-cytotoxic t-lymphocyte associated protein 4 (CTLA-4)], were included in both overall and progression free survival analysis using default KM Plotter settings (auto selection of best cutoff based on the calculation of all upper and lower quartiles with selection of the best performing threshold). For each gene of interest, a resulting KM plot was constructed with a reported HR, log-rank P-value, and FDR, with the y-axis representing probability of survival and the x-axis representing time (months). The total number of patients at risk for each time point is also reported. Similarly, an overall survival analysis was conducted for all chemokines of the 12-CK signature (75) using the same analysis parameters. Additionally, Spearman correlations between both *TCN2* and *CD320* with all chemokines of the 12-CK TLS signature including C-C Motif Chemokine Ligand (*CCL*)*2*, *CCL3*, *CCL4*, *CCL5*, *CCL8*, *CCL18*, *CCL19*, *CCL21*, *CXCL9*, *CXCL10*, *CXCL11*, *CXCL13* were also conducted. Due to an inherent limitation of the KM Plotter Immunotherapy database, a maximum of three genes of the 12-CK signature (in addition to either *TCN2* or *CD320*) were included within the same correlation analysis. Spearman correlation coefficients and P-values (* = P < 0.05, ** = P < 0.01, *** = P < 0.001, **** = P < 0.0001) were reported for all four-gene correlation matrices.

### 2.9 TCGA 12-CK signature correlation analysis

Using the Gene Expression Profiling Interactive Analysis 2 (GEPIA2) web server (http://gepia2.cancer-pku.cn/#index) (77), a multi-gene Spearman correlation analysis between either *TCN2* or *CD320* and the 12-CK TLS signature (75) was also performed. Two separate analyses using only the TCGA-SARC gene expression dataset or all tumor types of the TCGA were conducted. For each correlation analysis, the log2 of the transcript count per million [log2(TPM)] of the gene of interest (*TCN2* or *CD320*) was plotted on the x-axis while the log2(TPM) of the 12-CK gene signature is plotted on the y-axis. Importantly, while a log scale is used for graphical representation, the Spearman correlation is calculated on a non-log scale. The resulting Spearman correlation coefficients (R) and P-values are reported.

## 3 Results

### 3.1 Study design and patient stratification

Using the SomaLogic SomaScan® 7k assay, the plasma proteomic profile of primary, recurrent, and metastatic OSA patients was investigated. In total, 25 OSA patient plasma samples at various stages of disease and treatment status were processed in this analysis. Considering our group’s inherent interest in tumor immunology, two radiation-induced OSA patient samples, five active chemotherapy patient samples, and four post-chemotherapy patient samples were excluded from downstream analysis to mitigate confounding influences to the OSA proteome. The remaining samples, representing 14 naive OSA patients collected at diagnosis (before standard of care chemotherapeutic and/or surgery regimens), were then stratified into 2-year deceased and survivor all comers cohorts (**Figure 1C**). Patient information including sex, vital status, treatment, disease, and 2-year survival status, in addition to Streck-plasma spin down time (hours) from blood collection to plasma isolation and frozen storage, are reported (**Table 1**).

### 3.2 Proteomic assessment of naive OSA patients reveals DEPs associated with 2-year survival status

**Figure 2:**
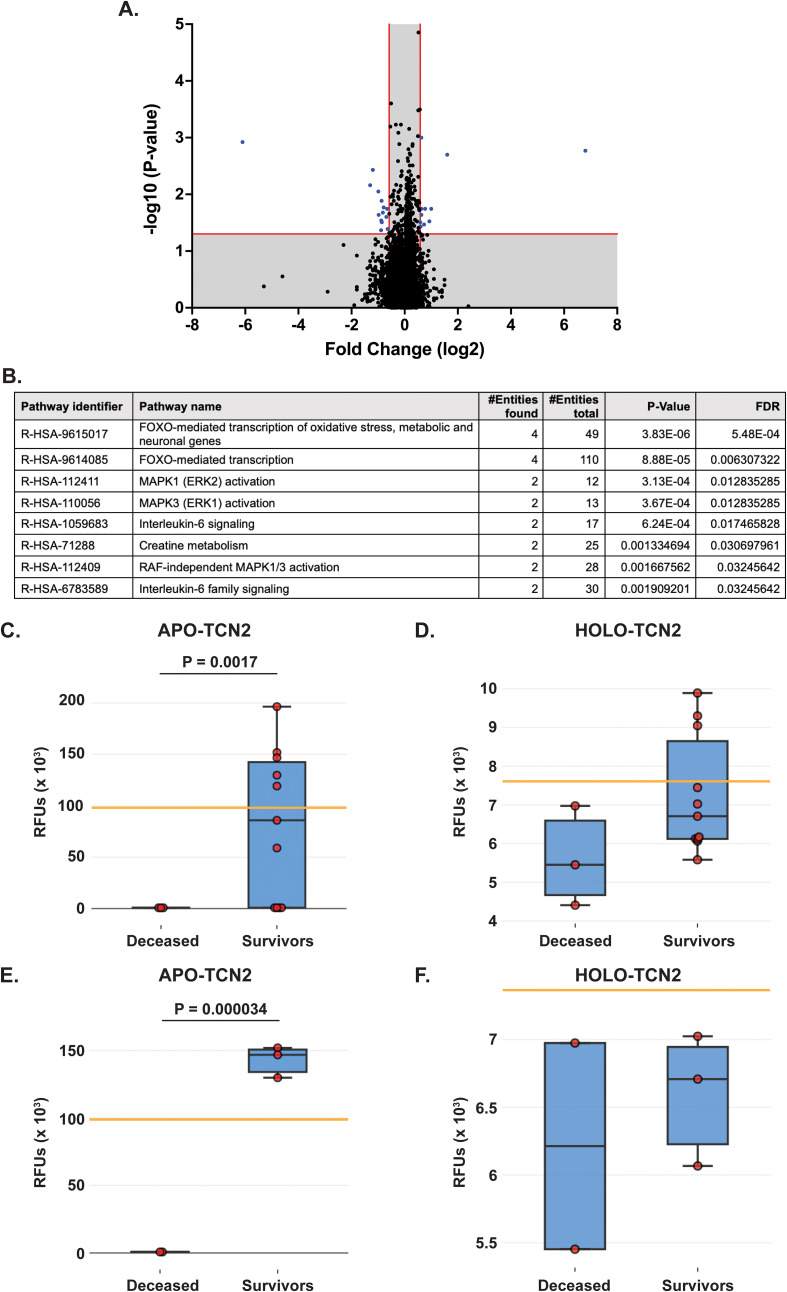
Proteomic analysis of naive OSA patients, stratified by 2-year survival status. **(A)** Volcano plot depicting those differentially expressed proteins (DEPs – blue) with a Log2FC > 0.585 or < -0.585 and P-value < 0.05. **(B)** Reactome analysis identifies those pathways associated with 2-year survival in OSA. Significant pathways include those with multiple entities and FDR < 0.05. Box plot depicting APO-TCN2 [seq.15560.52] **(C)** and HOLO-TCN2 [seq.5584.21] **(D)** levels in all naive OSA patients (n = 14) stratified by 2-year survival status, with n = 3 deceased and n = 11 survivors. Box plot depicting APO-TCN2 [seq.15560.52] **(E)** and HOLO-TCN2 [seq.5584.21] **(F)** levels in naive OSA patients with advanced disease (n = 5) stratified by 2-year survival status, with n = 2 deceased and n = 3 survivors. Each dot of the box plot is representative of an individual OSA patient. Y-axis represents the relative fluorescence units (RFUs) in thousands. Significant P-values (t-test) are presented for each comparison. Median values from assessment of plasma samples in a normal, healthy patient population (provided by SomaLogic, Inc.) are indicated by orange bar.

All experimental patient plasma samples processed at SomaLogic’s Boulder, CO facility passed standardized quality assessment and control (**Supplementary File 1** – **Supplementary Figure 1**). The plasma proteomic profiles of the 2-year deceased (n = 3) and survivor (n = 11) patients from an all comers OSA cohort (n = 14) were then compared (**Supplementary File 2**). Importantly, a total of 24 human DEPs were identified from this analysis, including 10 significantly upregulated and 14 significantly downregulated in the 2-year survivor cohort (**Figure 2A** and **Table 2**). These 24 DEPs were then inputted into the Reactome Pathway Analysis tool. The Reactome genome-wide overview map highlighted overrepresentation of these 24 DEPs in various pathways involved in the Immune System, Signal Transduction, and Metabolism (**Supplementary File 1** – **Supplementary Figure 2**). The most significant pathways (FDR < 0.05) and their enriched entities included those associated with FOXO-mediated transcription [catalase (CAT), resistin (RETN)], MAPK activation (IL-6R), Interleukin-6 signaling (IL-6R), and creatine metabolism [creatine kinase (CK)M, CKB] (**Figure 2B**).

**Table 2:** Differentially expressed proteins (DEPs) for the naive all OS 2-year survival comparison.

Sequence ID	Protein Name	UniProt ID	Gene Symbol	Log2FC	P-value
15560-52	Apo-Transcobalamin-2	P20062	TCN2	6.8	0.0017
19141-22	Death-associated protein 1	P51397	DAP	1.6	0.002
3714-49	Creatine kinase M-type:Creatine kinase B-type heterodimer	P12277|P06732	CKB|CKM	1	0.018
3488-64	Catalase	P04040	CAT	0.93	0.03
13534-20	Myomesin-2	P54296	MYOM2	0.73	0.034
11481-25	Hepatitis A virus cellular receptor 2	Q8TDQ0	HAVCR2	0.63	0.023
13691-10	Sodium-coupled monocarboxylate transporter 1	Q8N695	SLC5A8	0.63	0.018
4139-71	Interleukin-6 receptor subunit alpha	P08887	IL6R	0.63	0.001
15427-35	Lysyl oxidase homolog 3	P58215	LOXL3	0.6	0.037
15466-30	Collagen alpha-1(IX) chain	P20849	COL9A1	0.59	0.031
3216-2	Polymeric immunoglobulin receptor	P01833	PIGR	-0.64	0.041
13125-45	Vitronectin	P04004	VTN	-0.66	0.018
19323-1	Receptor-binding cancer antigen expressed on SiSo cells	O00559	EBAG9	-0.69	0.025
3199-54	Kallikrein-12	Q9UKR0	KLK12	-0.79	0.017
23569-53	START domain-containing protein 10	Q9Y365	STARD10	-0.82	0.021
12663-1	Thiosulfate sulfurtransferase	Q16762	TST	-0.86	0.031
3046-31	Resistin	Q9HD89	RETN	-0.87	0.013
17742-2	Ras-related protein R-Ras	P10301	RRAS	-0.88	0.029
13495-48	Hydroxycarboxylic acid receptor 2	Q8TDS4	HCAR2	-0.89	0.043
23259-23	EEF1A lysine methyltransferase 1	Q8WVE0	EEF1AKMT1	-0.98	0.023
4964-67	Endoplasmic reticulum aminopeptidase 1	Q9NZ08	ERAP1	-0.99	0.0089
15388-24	Low affinity immunoglobulin gamma Fc region receptor III-A	P08637	FCGR3A	-1.2	0.0037
12581-39	Inositol monophosphatase 2	O14732	IMPA2	-1.3	0.0069
10966-1	Alpha-2-HS-glycoprotein	P02765	AHSG	-6.1	0.0012

Of the 24 individual DEPs, APO-TCN2 was the most significantly (P = 0.0017) upregulated protein associated with 2-year survival, reporting a Log2FC = 6.8. For n = 5 of total 11 patients in the 2-year survivor cohort, APO-TCN2 levels vastly exceeded median normal patient plasma RFU measurements (provided by SomaLogic) (58), with an additional two patients reporting well above minimal detectable levels. This finding contrasts with all three deceased patients, which measured near minimal detectable levels (**Figure 2C**). Interestingly, while the vitamin-B12 unbound form of Transcobalmin-2 (APO-TCN2) differed vastly between 2-year deceased and survivor cohorts, no difference in the vitamin-B12 bound form (HOLO-TCN2) was measured (**Figure 2D**). Importantly, the lack of measurable differences in HOLO-TCN2 levels suggests that these OSA patients, likely, do not have a genetic *TCN2* alteration or abnormality, as previously reported in various case reports (78–81). Of note, a significant (P = 0.0012) decrease in Alpha-2-HS-glycoprotein (AHSG) was also associated with 2-year survival status, reporting a log2FC = -6.1 (**Table 2**). Furthermore, a secondary analysis comparing the proteome of 2-year deceased (n = 2) and survivors (n = 3) of the Advanced Disease sub-cohort (n = 5) further supported these findings. A significant (P = 0.000034) increase in plasma APO-TCN2 (**Figure 2E**), the most differentially upregulated protein in 2-year survivors, and no difference in HOLO-TCN2 levels (**Figure 2F**), was again associated with 2-year survival status. Similarly, AHSG levels were significantly (P = 0.026) reduced in the 2-year survival cohort (**Supplementary File 3**). Overall, a comparative proteomic analysis of OSA patient peripheral plasma identified 24 DEPs associated with 2-year survival status. Of those proteins, both APO-TCN2 and AHSG measured vastly increased or decreased levels in the survivor cohort, respectively. To further elucidate both proteins’ relevance in OSA and other solid tumors, publicly available bulk and scRNA-seq tumor datasets were then examined.

### 3.3 Survival Genie analysis reveals association of elevated *TCN2* with overall survival in sarcoma

**Figure 3:**
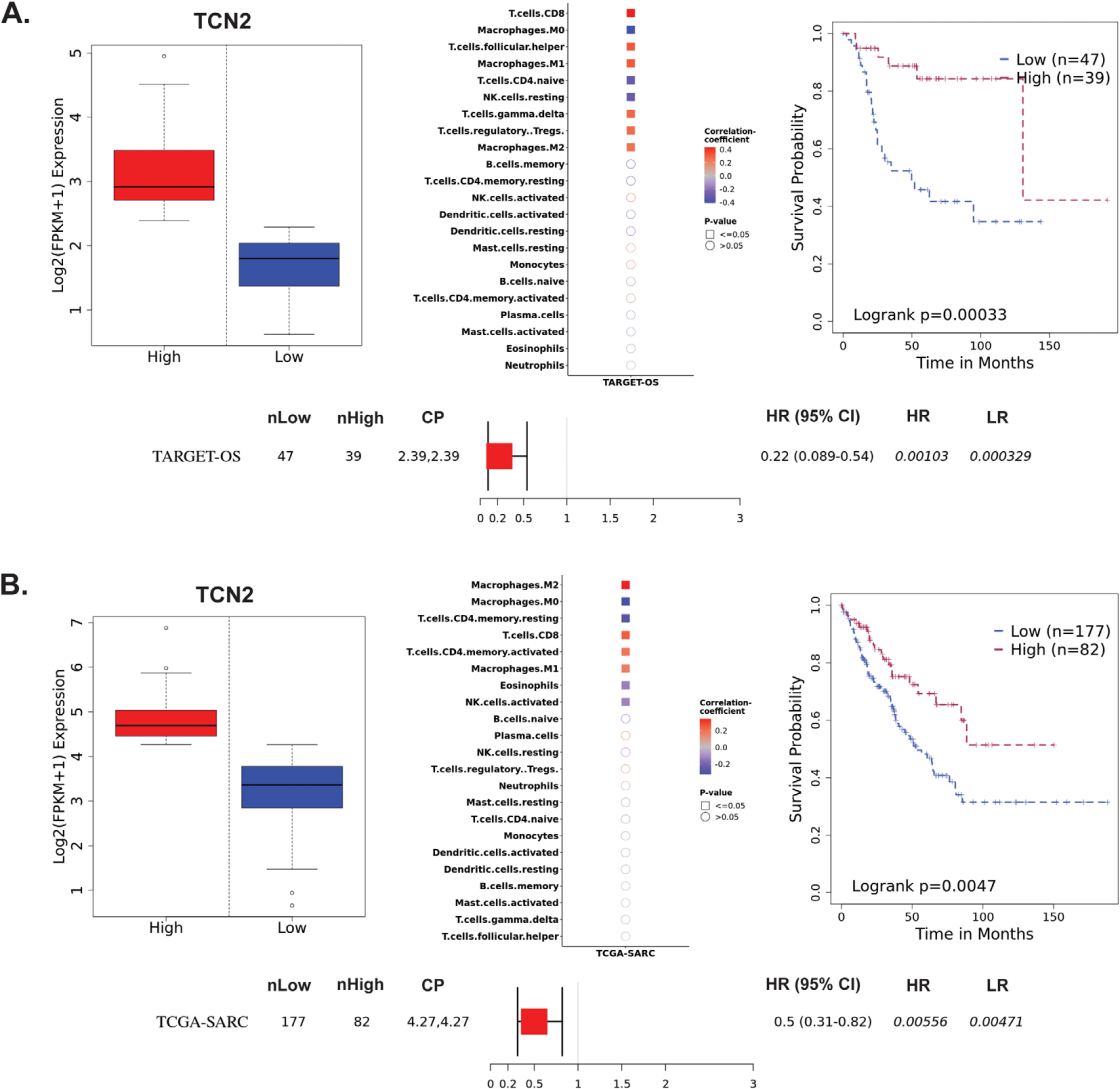
TARGET-OS and TCGA-SARC survival analysis supports TCN2 as a marker of better overall survival. Analysis of *TCN2* in TARGET-OS **(A)** and TCGA-SARC **(B)** databases using Survival Genie web-based platform. Box plots representing *TCN2* FPKM normalized expression in primary tumors with stratification into low and high expressing groups. The relative fraction of TILs was estimated using the tumor-infiltrating immune cell type matrix LM22 gene signature and CIBERSORTx deconvolution. Pearson correlation matrix of deconvoluted immune cell RNA-seq gene expression data and *TCN2*, with shape (square or circle) denoting significance and color denoting positive (red) or negative (blue) correlation with *TCN2*. Kaplan-Meier (KM) survival curves with high (red) and low (blue) group stratification are compared using the log-rank test, with log-rank P-value < 0.05 considered statistically significant. Forest plot details the association between the high and low groups [stratified by cut point (CP)] based on the Cox Proportional Hazards regression model. nLow and nHigh represent the number of patients in low and high expressing groups, respectively. Hazard ratio (HR) with 95% confidence interval as well as the associated Wald-test (HR) and log-rank (LR) P-values are reported.

The Survival Genie web-based platform was then used to associate expression of *TCN2* and *AHSG* with overall survival in publicly available OSA (TARGET-OS) and sarcoma (TCGA-SARC) bulk tumor RNA-sequencing datasets (62). To begin, evaluation of *TCN2* expression in the TARGET-OS database identified 47 patients with low and 39 patients with high *TCN2* expression in OSA primary tumors. Using CIBERSORTx (65), a significant (P ≤ 0.05) positive correlation between *TCN2* expression and T.cells.CD8, T.cells.follicular.helper, Macrophages.M1, T.cells.gamma.delta, T.cells.regulatory.Tregs, and Macrophages.M2 gene signatures was revealed. Additionally, a significant (P ≤ 0.05) negative correlation between *TCN2* expression and Macrophages.M0, T.cells.CD4.naive, and NK.cells.resting gene signatures was also apparent. Importantly, Kaplan-Meier survival curve analysis associated an increase in intratumoral *TCN2* expression with better overall survival (log-rank P= 0.00033), with a reported HR = 0.22 (0.089 – 0.54) (**Figure 3A**). Considering a shared sarcomatous background, our group expanded the analysis to assess *TCN2* gene expression in the larger TCGA-SARC dataset to provide rigor and support to the experimental findings uncovered in TARGET-OS. Analysis of the TCGA-SARC dataset identified 177 patients with low and 82 patients with high expression of *TCN2* in sarcoma primary tumors. Using CIBERSORTx (65), a significant (P ≤ 0.05) positive correlation between *TCN2* expression and Macrophages.M2, T.cells.CD8, T.cells.CD4.memory.activated, and Macrophages.M1 was identified. Additionally, a significant (P ≤ 0.05) negative correlation between *TCN2* expression and Macrophages.M0, T.cells.CD4.memory.resting, Eosinophils, and NK.cells.activated gene signatures was also apparent. Importantly, Kaplan-Meier curve survival analysis again associated an increase in *TCN2* expression with better overall survival (log-rank P = 0.0047), with a reported HR = 0.5 (0.31 – 0.82) (**Figure 3B**). These results suggest that increased intratumoral *TCN2* is associated with an activated immune signature and better overall survival. While a similar analysis revealed that low *AHSG* expression was associated with better overall survival in the TARGET-OS dataset (corroborating our proteomic findings), this trend was not apparent in the TCGA-SARC analysis, and neither finding was statistically significant. Therefore, AHSG was excluded from further downstream analysis (**Supplementary File 1** – **Supplementary Figure 3**). Overall, Kaplan-Meier survival curve analysis associated elevated *TCN2* expression with significantly improved outcomes in both the TARGET-OS and TCGA-SARC datasets. Importantly, the association of increased intratumoral *TCN2* expression with better overall survival correlated with our previous plasma proteomics findings (**Figures 2B, D**). These results ultimately support both peripheral blood plasma APO-TCN2 and intratumoral *TCN2* gene expression as possible biomarkers of overall survival in this disease.

### 3.4 TIGER reveals expression patterns of *TCN2* in solid tumors

**Figure 4:**
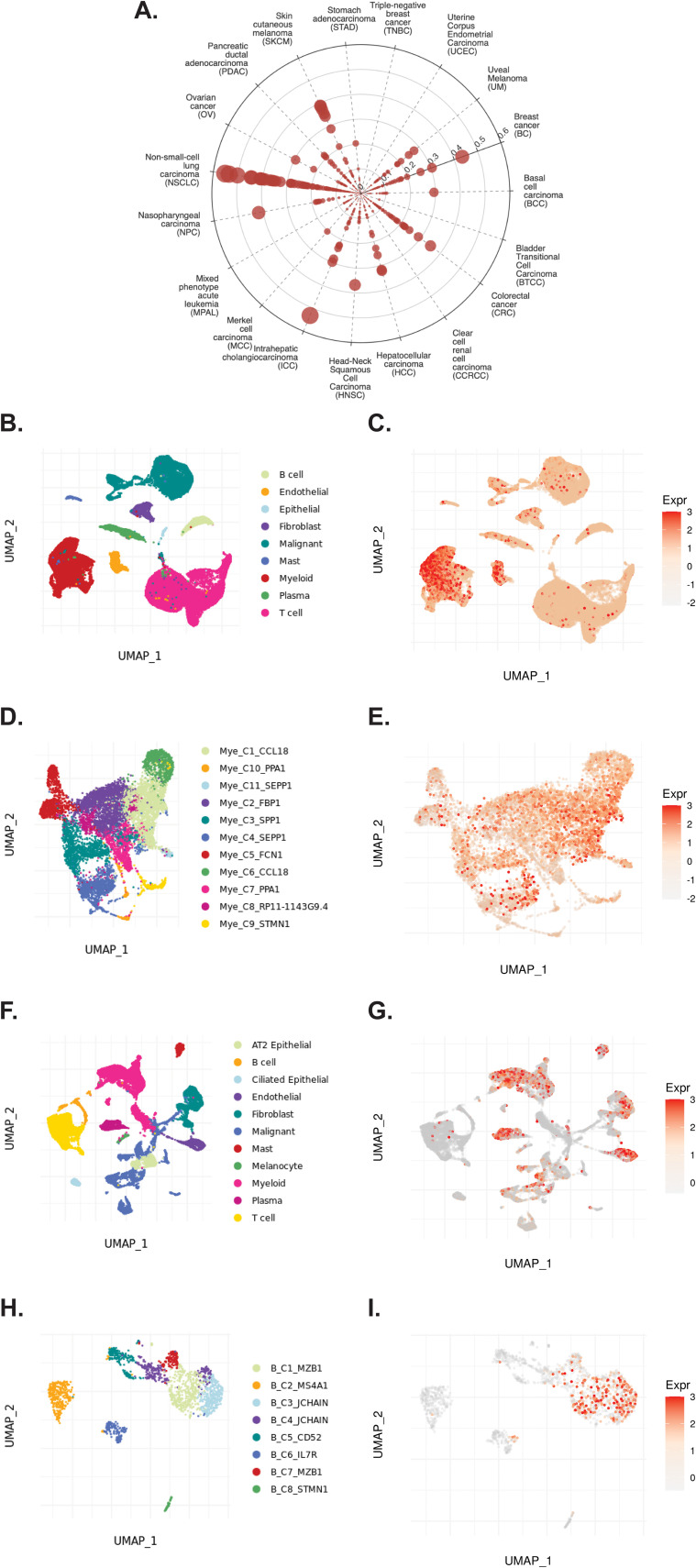
TIGER database confirms *TCN2* expression on various myeloid and B cellular clusters of NSCLC datasets. **(A)** Cell Type Marker (Log2FC) diagram represents differential expression of *TCN2* on various clusters and subclusters in each scRNA-seq tumor dataset of the TIGER database, with differential expression represented by circle size and distance from center. **(B, C)** UMAPs of all cellular clusters of the NSCLC dataset highlight expression of *TCN2* on myeloid and endothelial cells. **(D, E)** UMAPs of the Myeloid subclusters of the NSCLC dataset highlighting expression of *TCN2*. **(F, G)** UMAPs of all cellular clusters of the NSCLC6 dataset highlights expression of *TCN2* on endothelial and plasma cells. **(H, I)** UMAPs of the B cell sub-clusters of the NSCLC6 dataset highlighting expression of *TCN2*. UMAPs generated using the TIGER portal.

The TIGER portal was then used to investigate the expression of *TCN2* across identified cellular clusters of various solid tumor scRNA-seq datasets (66). While previous analyses of bulk transcriptomic data through CIBERSORTx deconvolution had suggested the likely cellular source for *TCN2* gene expression in sarcomatous tumors, our group sought to determine which cells differentially expressed *TCN2* (for a variety of tumor types) using a method that provides single cell resolution to the complex and heterogenous tumor microenvironment (TME). Numerous cellular clusters across many solid tumors revealed differential expression of *TCN2* including non-small cell lung cancer (NSCLC), intrahepatic cholangiocarcinoma (ICC), breast cancer (BC), nasopharyngeal carcinoma (NPC), and skin cutaneous melanoma (SKCM), among others (**Figure 4A**). The top 20 cellular clusters with the greatest increase in *TCN2* expression were reported, highlighting apparent increases for B cell, Endothelial, and Myeloid cellular clusters, particularly in both NSCLC and SKCM datasets (**Table 3**). To visualize these expression changes, subsequent analysis of Uniform Manifold Approximation and Projections (UMAPs) and gene expression boxplots for the NSCLC dataset (67) confirmed isolated increases in *TCN2* expression on both Myeloid and Endothelial cellular clusters (**Figures 4B, C**). Subcluster analysis of the Myeloid cells revealed near ubiquitous expression of *TCN2* across myeloid populations, with the greatest increases apparent for the Mye_C1_CCL18 and Mye_C6_CCL18 subclusters (**Figures 4D, E**). Further visualization of the NSCLC6 (70) dataset supported increased *TCN2* expression on Endothelial, Plasma, AT2 Epithelial, and Myeloid cell populations (**Figures 4F, G**). Additional subcluster analysis of B cells revealed increased expression across B_C3_JCHAIN, B_C1_MZB1, and B_C7_MZB1 cellular clusters (**Figures 4H, I**), in accordance with the findings highlighted in **Table 3**. Similar results were also apparent for the SKCM1 dataset (71), which revealed increased *TCN2* expression on all Myeloid cells, various Myeloid cell subclusters including Mye_C4_C1QA, as well as the B_C2_IGHG1 B cell subcluster (**Supplementary File 1** – **Supplementary Figure 4**). Overall, these data corroborated our Survival Genie CIBERSORTx data and previous literature (82) which suggested that *TCN2* is mainly expressed (in immune cells) on Myeloid populations.

**Table 3:** Top 20 clusters with increased expression of *TCN2* within the TIGER database.

Cancer Type	Dataset ID	Cell Lineage	Cell Type	Log2FC
Non-small-cell lung carcinoma (NSCLC)	NSCLC6	B cell	B_C3_JCHAIN	0.5526
Non-small-cell lung carcinoma (NSCLC)	NSCLC6	All	Endothelial	0.5362
Intrahepatic cholangiocarcinoma (ICC)	ICC	All	Endothelial	0.5315
Non-small-cell lung carcinoma (NSCLC)	NSCLC	Myeloid	Mye_C1_CCL18	0.5022
Non-small-cell lung carcinoma (NSCLC)	NSCLC6	B cell	B_C1_MZB1	0.4472
Breast cancer (BC)	BC	Myeloid	Mye_C3_C1QC	0.4345
Nasopharyngeal carcinoma (NPC)	NPC	Myeloid	Mye_C2_CTSD	0.4186
Non-small-cell lung carcinoma (NSCLC)	NSCLC6	All	Plasma	0.4098
Non-small-cell lung carcinoma (NSCLC)	NSCLC5	Myeloid	Mye_C7_APOE	0.4009
Non-small-cell lung carcinoma (NSCLC)	NSCLC	All	Myeloid	0.3911
Skin cutaneous melanoma (SKCM)	SKCM1	B cell	B_C2_IGHG1	0.3895
Skin cutaneous melanoma (SKCM)	SKCM1	Myeloid	Mye_C4_C1QA	0.3865
Head-Neck Squamous Cell Carcinoma (HNSC)	HNSC	Myeloid	Mye_C7_APOE	0.3681
Non-small-cell lung carcinoma (NSCLC)	NSCLC3	Myeloid	Mye_C3_CTSB	0.357
Skin cutaneous melanoma (SKCM)	SKCM1	All	Myeloid	0.3546
Non-small-cell lung carcinoma (NSCLC)	NSCLC1	Myeloid	Mye_C6_CTSB	0.3541
Colorectal cancer (CRC)	CRC1	Myeloid	Mye_C3_APOE	0.3505
Non-small-cell lung carcinoma (NSCLC)	NSCLC6	Myeloid	Mye_C2_APOE	0.3453
Skin cutaneous melanoma (SKCM)	SKCM1	All	Plasma	0.3427
Hepatocellular carcinoma (HCC)	HCC	All	Endothelial	0.3222

### 3.5 TIGER reveals expression patterns of *CD320* in solid tumors

**Figure 5:**
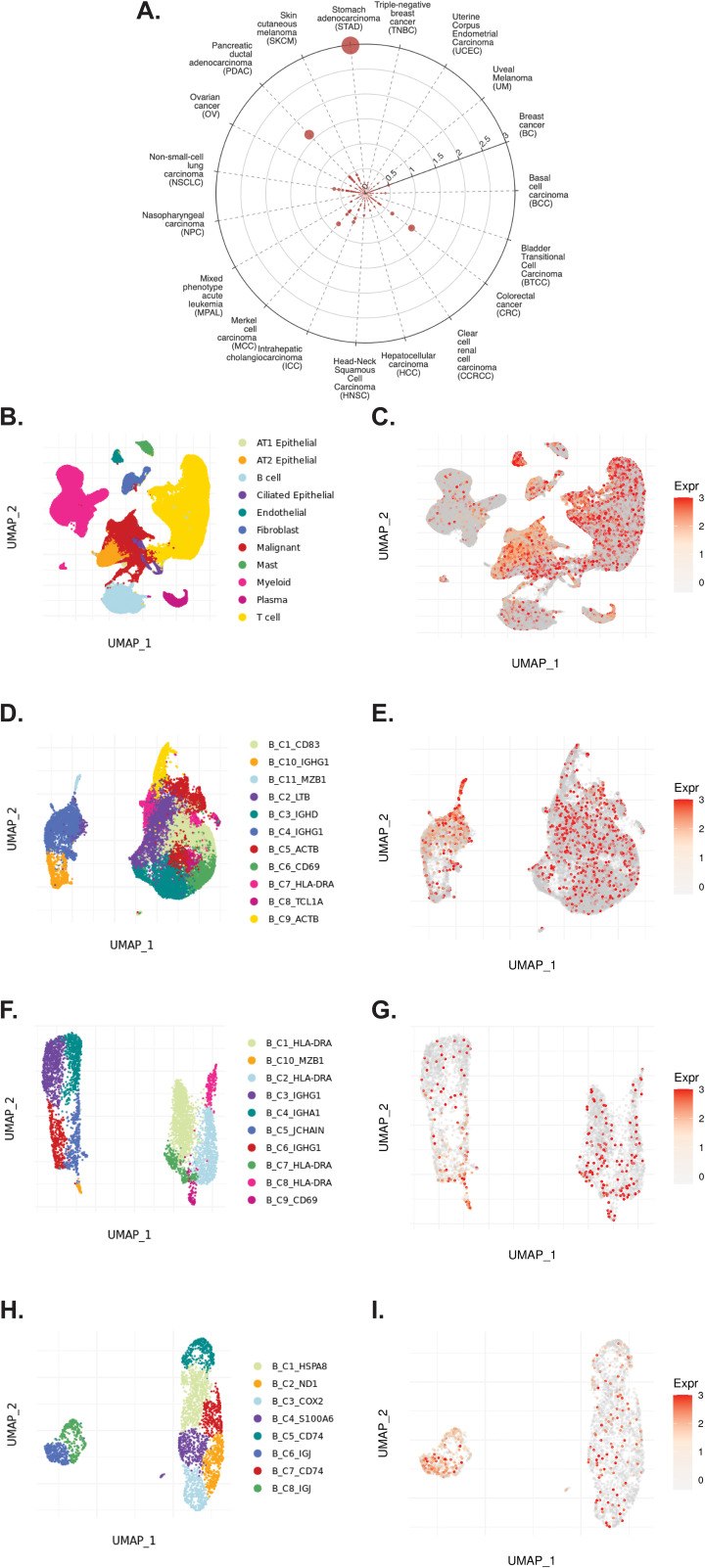
TIGER database confirms *CD320* expression on various myeloid and B cellular clusters of NSCLC datasets. **(A)** Cell Type Marker (Log2FC) diagram represents differential expression of *CD320* on various clusters and subclusters in each scRNA-seq tumor dataset of the TIGER database, with differential expression represented by circle size and distance from center. **(B, C)** UMAPs of all cellular clusters of the NSCLC5 dataset highlights expression of *CD320* on endothelial, malignant, and fibroblast cellular clusters. **(D, E)** UMAPs of the B cell subclusters of the NSCLC5 dataset highlighting expression of *CD320*. **(F, G)** UMAPs of B cell subclusters of the NSCLC dataset highlighting expression of *CD320*. **(H, I)** UMAPs of B cell subclusters of the NSCLC1 dataset highlighting expression of *CD320.* UMAPs generated using the TIGER portal.

The TIGER portal was also used to investigate the expression of *CD320* (83), the TCN2 receptor, across identified cellular clusters of solid tumor scRNA-seq datasets (66). Numerous cellular clusters across many solid tumors revealed differential expression of *CD320* including stomach adenocarcinoma (STAD), colorectal cancer (CRC), Merkel cell carcinoma (MCC), NSCLC, ICC, and SKCM, among others (**Figure 5A**). The top 20 cellular clusters with the greatest increase in *CD320* expression were reported, highlighting apparent increases for Endothelial, Malignant, B cell, CD4, and Myeloid cellular clusters across a variety of solid tumors (**Table 4**). To visualize these expression changes on immune cells of interest (B and Myeloid cells), subsequent analysis of UMAPs and gene expression boxplots for the NSCLC5 dataset confirmed increases in *CD320* expression on Endothelial, Malignant, and Fibroblast cellular clusters (**Figures 5B, C**). Additional subcluster analysis of B cells supported an increase in *CD320* expression on both B_C11_MZB1 and B_C4_IGHG1 (**Figure 5D, E**). Further visualization of UMAPs and gene expression boxplots for B cell subclusters of both the NSCLC (**Figures 5F, G**) and NSCLC1 (**Figures 5H, I**) datasets supported increased *CD320* gene expression on various Marginal Zone B and B1 Cell Specific Protein (*MZB1)+* and *IGJ+* B cell subclusters, in accordance with findings from **Table 4**. Similar results were also apparent for the SKCM1 dataset, which revealed increased *CD320* expression on all Plasma cells, Myeloid cell subclusters including Mye_C1_GZMB, as well as the B_C2_IGHG1 B cell subcluster (**Supplementary File 1** – **Supplementary Figure 5**). Overall, these data corroborated previous publications which suggested that while *CD320* is expressed ubiquitously across many cell types (84), its expression is increased on proliferating cells (85), such as malignant cells or B lymphocytes (86), in a variety of solid tumors (87).

**Table 4:** Top 20 clusters with increased expression of *CD320* within the TIGER database.

Cancer Type	Dataset ID	Cell Lineage	Cell Type	Log2FC
Stomach adenocarcinoma (STAD)	STAD	All	Endothelial	2.989
Pancreatic ductal adenocarcinoma (PDAC)	PDAC	All	Endothelial	1.6355
Colorectal cancer (CRC)	CRC1	All	Endothelial	1.1556
Merkel cell carcinoma (MCC)	MCC	All	Malignant	0.8158
Colorectal cancer (CRC)	CRC2	All	Endothelial	0.6713
Non-small-cell lung carcinoma (NSCLC)	NSCLC5	B cell	B_C11_MZB1	0.6342
Intrahepatic cholangiocarcinoma (ICC)	ICC	B cell	B_C5_HIST1H4C	0.6209
Non-small-cell lung carcinoma (NSCLC)	NSCLC	B cell	B_C10_MZB1	0.5401
Intrahepatic cholangiocarcinoma (ICC)	ICC	All	Endothelial	0.5392
Breast cancer (BC)	BC	All	Endothelial	0.4935
Pancreatic ductal adenocarcinoma (PDAC)	PDAC2	CD4	CD4_C10_GNLY	0.4653
Non-small-cell lung carcinoma (NSCLC)	NSCLC6	All	Endothelial	0.4624
Pancreatic ductal adenocarcinoma (PDAC)	PDAC2	All	Endothelial	0.4548
Head-Neck Squamous Cell Carcinoma (HNSC)	HNSC	All	Erythrocyte	0.4403
Skin cutaneous melanoma (SKCM)	SKCM1	Myeloid	Mye_C1_GZMB	0.4134
Skin cutaneous melanoma (SKCM)	SKCM1	B cell	B_C2_IGHG1	0.4074
Basal cell carcinoma (BCC)	BCC	Myeloid	Mye_C1_GZMB	0.4006
Non-small-cell lung carcinoma (NSCLC)	NSCLC1	B cell	B_C6_IGJ	0.3837
Non-small-cell lung carcinoma (NSCLC)	NSCLC5	All	Endothelial	0.3828
Colorectal cancer (CRC)	CRC2	Myeloid	Mye_C13_GZMB	0.3781

### 3.6 TIGER cell-cell communication analysis reveals interaction between *TCN2*+ myeloid cells and *CD320*+ B cells in solid tumors

To investigate a possible interaction between *TCN2*+ Myeloid and *CD320+* B cells in these solid tumors, the TIGER portal was then used to perform a cell-cell communication analysis (66). Further investigation of both the NSCLC (67) and SKCM1 (71) datasets was warranted considering the increased expression of both *TCN2* (ligand) and *CD320* (receptor) on various immune cell populations of these tumors. To begin, a cell-cell communication analysis for the Mye_C1_CCL18 subcluster, with the greatest increase in *TCN2* expression of all myeloid cells of the NSCLC dataset (67), was performed. Analysis revealed that Mye_C1_CCL18 had greatest interaction with various other myeloid subclusters including Mye_C2_FBP1 (effect score = 97.684), Mye_C7_PPA1 (effect score = 97.64), and Mye_C6_CCL18 (effect score = 96.877). The top 20 receptor-ligand interactions driving these effect scores were reported (**Table 5**). Of note, this analysis consistently highlighted likely receptor-ligand interactions between CD74_MIF, CD74_COPA, CD74_APP, and HLA-DPB1_TNFSF13B as well as additional communication through HLA-DRB1_TNFSF9, CCR1_CCL18, and TNFRSF1A_GRN (**Table 5**). Similar cell-cell communication patterns were evident for the Mye_C4_C1QA subcluster of the SKCM1 dataset (71), which displayed the greatest increase in *TCN2* expression of all SKCM1 myeloid subclusters. Here, Mye_C4_C1QA also reported the greatest cell-cell communication effects scores with other myeloid subclusters (**Supplementary File 1** – **Supplementary Table 1**).

**Table 5:** Top 3 interactions defined by cell-cell communication analysis for Mye_C1_CCL18 of the NSCLC dataset (TIGER), with effect score presented in parenthesis.

Gene | Gene	Mye_C2_FBP1 | Mye_C1_CCL18 (97.684)	Gene | Gene	Mye_C7_PPA1 | Mye_C1_CCL18 (97.64)	Gene | Gene	Mye_C6_CCL18 | Mye_C1_CCL18 (96.877)
CD74_MIF	2.69	CD74_MIF	3.075	CD74_MIF	2.819
CD74_COPA	2.41	CD74_COPA	2.795	HLA-DPB1_TNFSF13B	2.607
CD74_APP	2.379	CD74_APP	2.765	CD74_COPA	2.539
HLA-DPB1_TNFSF13B	2.186	HLA-DPB1_TNFSF13B	2.495	CD74_APP	2.509
HLA-DRB1_OGN	1.861	HLA-DRB1_OGN	2.111	HLA-DPA1_TNFSF9	2.016
C5AR1_RPS19	1.809	HLA-DPA1_TNFSF9	2.015	HLA-DRB1_OGN	1.864
CCR1_CCL18	1.775	C5AR1_RPS19	1.695	C5AR1_RPS19	1.798
HLA-DPA1_TNFSF9	1.605	CCR1_CCL18	1.689	HLA-C_FAM3C	1.725
SPP1_CD44	1.529	HLA-C_FAM3C	1.594	CCR1_CCL18	1.617
HLA-C_FAM3C	1.528	TNFRSF1A_GRN	1.489	TNFRSF1A_GRN	1.43
TNFRSF1A_GRN	1.46	TNFRSF1B_GRN	1.417	ANXA1_FPR3	1.324
TNFRSF1B_GRN	1.361	SPP1_CD44	1.278	ANXA1_FPR1	1.275
GRN_SORT1	1.089	EGFR_GRN	1.222	GRN_SORT1	1.27
LGALS9_CD44	1.059	GRN_SORT1	1.22	TNFRSF1B_GRN	1.255
ANXA1_FPR3	1.043	LGALS9_CD44	1.086	LGALS9_CD44	1.172
CD52_SIGLEC10	1.022	HLA-E_KLRC1	1.018	ANXA1_FPR2	1.102
SPP1_PTGER4	1.01	PTPRC_MRC1	0.978	CD52_SIGLEC10	1.097
ANXA1_FPR1	0.993	ANXA1_FPR3	0.961	ALOX5_ALOX5AP	1.089
SPP1_a4b1 complex	0.979	CD94:NKG2A_HLA-E	0.96	LGALS9_CD47	1.006
HLA-E_KLRC1	0.963	NRG1_MS4A4A	0.931	CD99_PILRA	0.991

Top 20 receptor-ligand interactions (gene | gene) driving the overall effect score between interacting cellular clusters.

Additionally, a cell-cell communication analysis for the B_C10_MZB1 subcluster, with the greatest increase in *CD320* expression of all B cells of the NSCLC dataset (67), was performed. Analysis revealed that B_C10_MZB1 had the greatest interaction with various myeloid subclusters including Mye_C1_CCL18 (effect score = 48.993), Mye_C2_FBP1 (effect score = 47.944), and Mye_C7_PPA1 (effect score = 46.079). The top 20 receptor-ligand interactions driving these elevated effect scores were reported. Of note, this analysis consistently highlighted likely receptor-ligand interactions between CD74_MIF, CD74_COPA, CD74_APP, and HLA-C_FAM3C as well as additional communication through C5AR1_RPS19, HLA-DPB1_TNFSF13B, CD94:NKG2A_HLA-E, SPP1_CD44, and various chemokine axes (**Table 6**). Similar cell-cell communication patterns were also evident for the B_C2_IGHG1 subcluster of the SKCM1 dataset (71), which displayed the greatest increase in *CD320* expression of all SKCM1 B cell subclusters. Importantly, the B_C2_IGHG1 subcluster displayed the greatest cell-cell communication effect score with various myeloid subclusters including Mye_C4_C1QA (**Supplementary File 1** – **Supplementary Table 2**). Overall, cell-cell communication analysis revealed that those B cell subclusters with greatest expression of *CD320* [B_C10_MZB1 (NSCLC), B_C2_IGHG1 (SKCM1)] reported the greatest cell-cell communication effect scores with those Myeloid cell subclusters with greatest expression of *TCN2* [Mye_C1_CCL18 (NSCLC), Mye_C4_C1QA (SKCM1)]. These results suggest a possible Myeloid (*TCN2*) to B/Plasma cell (*CD320*) communication network in the tumor microenvironment of immunogenic solid tumors. Considering TCN2’s previously described biological functions (88–90), it was hypothesized that this interaction in solid tumors would allow for enhanced B lymphocyte infiltration, proliferation, and germinal center/TLS formation (91).

**Table 6:** Top 3 interactions defined by cell-cell communication analysis for B_C10_MZB1 of the NSCLC dataset (TIGER), with effect score presented in parenthesis.

Gene | Gene	Mye_C1_CCL18 | B_C10_MZB1 (48.993)	Gene | Gene	Mye_C2_FBP1 | B_C10_MZB1 (47.944)	Gene | Gene	Mye_C7_PPA1 | B_C10_MZB1 (46.079)
CD74_MIF	2.983	CD74_MIF	2.906	CD74_MIF	3.291
CD74_COPA	2.491	CD74_COPA	2.414	CD74_COPA	2.799
CD74_APP	2.419	CD74_APP	2.342	CD74_APP	2.727
C5AR1_RPS19	2.121	C5AR1_RPS19	2.105	HLA-DPB1_TNFSF13B	2.044
HLA-DPB1_TNFSF13B	1.873	HLA-C_FAM3C	1.758	HLA-DPA1_TNFSF9	2.018
HLA-C_FAM3C	1.777	HLA-DPB1_TNFSF13B	1.735	C5AR1_RPS19	1.991
HLA-DPA1_TNFSF9	1.732	HLA-DPA1_TNFSF9	1.607	HLA-C_FAM3C	1.824
CD94:NKG2A_HLA-E	0.892	SPP1_CD44	1.104	CD94:NKG2A_HLA-E	0.893
SPP1_CD44	0.757	SPP1_a4b1 complex	1.004	SPP1_CD44	0.853
ANXA1_FPR3	0.689	SPP1_PTGER4	0.952	LGALS9_CD47	0.77
PLD2_ARF1	0.679	CD94:NKG2C_HLA-E	0.889	SPP1_a4b1 complex	0.754
LGALS9_CD47	0.664	PLAUR_a4b1 complex	0.813	MIF_TNFRSF14	0.717
SPP1_a4b1 complex	0.658	MIF_TNFRSF14	0.796	SPP1_PTGER4	0.701
LAMP1_FAM3C	0.628	ANXA1_FPR3	0.766	ANXA1_FPR3	0.684
PECAM1_CD38	0.622	LGALS9_CD47	0.744	PLD2_ARF1	0.677
CD44_HBEGF	0.621	CXCL10_CXCR3	0.74	LGALS9_CD44	0.66
EGFR_MIF	0.609	CD44_HBEGF	0.687	LGALS9_SLC1A5	0.647
CXCL12_CXCR4	0.606	PLD2_ARF1	0.67	PTPRC_CD22	0.61
SPP1_PTGER4	0.606	LGALS9_CD44	0.634	EGFR_MIF	0.604
PLAUR_a4b1 complex	0.588	CCL2_CCR10	0.624	CXCL12_CXCR4	0.587

Top 20 receptor-ligand interactions (gene | gene) driving the overall effect score between interacting cellular clusters.

### 3.7 scRNA-seq of OSA tumors supports presence of *TCN2*+ myeloid and *CD320*+ plasmacytes/B cells

**Figure 6:**
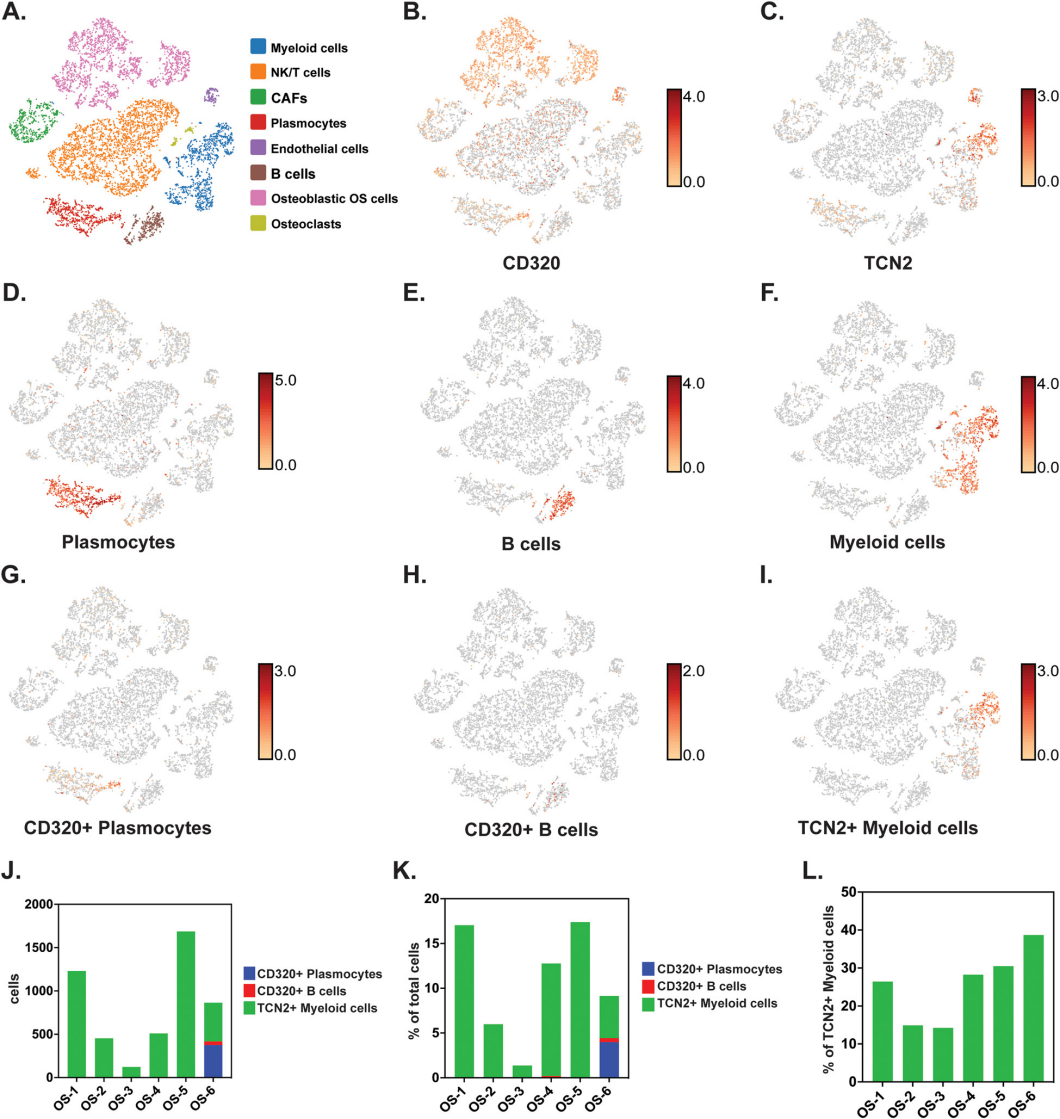
scRNA-seq analysis of naive primary OSA samples reveals *CD320* and *TCN2* expression. All clustering representative of patient OS-6 **(A)** All cellular clusters identified by canonical marker expression. **(B)** Representative log-normalized *CD320* expression across various cellular clusters. **(C)** Representative log-normalized *TCN2* expression across various cellular cluster of patient OS-6. **(D)** Representative clustering of Plasmocytes with log-normalized expression of *IGHG1* and *MZB1* using the “Feature Min” function. **(E)** Representative clustering of B cells with log-normalized expression of *MS4A1* and *CD79A* using the “Feature Min” function. **(F)** Representative clustering of Myeloid cells with log-normalized expression of *LYZ* and *CD68* using the “Feature Min” function. **(G)** Representative clustering of *CD320+* Plasmocytes with log-normalized expression of *CD320, IGHG1*, and *MZB1* using the “Feature Min” function. **(H)** Representative clustering of *CD320+* B cells with log-normalized expression of CD320, *MS4A1*, and *CD79A* using the “Feature Min” function. **(I)** Representative clustering of *TCN2+* Myeloid cells with log-normalized expression of *TCN2, LYZ*, and *CD68* using the “Feature Min” function. **(J)** Quantification of total cells for the *CD320+* Plasmocytes, *CD320+* B cells, and *TCN2+* Myeloid cells populations in all naive OSA patient tumors (n = 6). **(K)** Quantification of the percent (%) of total cells for *CD320+* Plasmocytes, *CD320+* B cells, and *TCN2+* Myeloid cells population in all naive OSA patient tumors (n = 6). **(L)** Quantification of the percent (%) of *TCN2+* Myeloid cells, relative to all Myeloid cells in all naive OSA patient tumors (n = 6).

To investigate whether *TCN2+* Myeloid and *CD320+* B Lymphocyte clusters existed in OSA tumors, and whether an increase in *TCN2+* Myeloid cells supports B lymphocyte infiltration and proliferation, scRNA-seq data from naive primary (GSE162454) and chemotherapy treated primary, recurrent, and metastatic (GSE152048) OSA tumors were analyzed. First, scRNA-seq analysis of six naive OSA primary tumors (GSE162454) was conducted. Each major cellular cluster was identified according to expression of canonical markers previously detailed by Liu et al. (72) (**Figure 6A**). Visualization of t-SNE plots revealed ubiquitous expression of *CD320* in OSA primary tumors, with greatest expression on Osteoblastic OSA cells, Endothelial cells, NK/T cells, and Plasmocytes (**Figure 6B**). For *TCN2*, t-SNE plots conveyed concentrated gene expression on Myeloid cells, with additional positivity on both Endothelial cells and Plasmocytes (**Figure 6C**). The number of Plasmocytes (**Figure 6D**), B cells (**Figure 6E**), and Myeloid cells (**Figure 6F**) expressing *CD320* (Plasmocytes/B cells, **Figures 6G, H**) or *TCN2* (Myeloid cells, **Figure 6I**) was then quantified. Unfortunately, only the OS-6 patient sample contained elevated levels of Plasmocyte or B cell populations (**Supplementary File 1** – **Supplementary Figure 6**). This finding was unsurprising considering Li et al. noted that only one patient sample had identifiable TLSs during sample processing (72). Nevertheless, total *CD320*+ Plasmocytes, *CD320*+ B cells, and *TCN2*+ Myeloid cells, as well as the percent (%) of total cells, were quantified for each patient sample (**Figures 6J, K**). Importantly, while only OS-6 contained a substantial number of *CD320+* Plasmocytes and B cells, this primary tumor also measured the greatest percentage (%) of *TCN2*+ Myeloid cells (relative to all Myeloid cells) at 38.68% (**Figure 6L** and **Supplementary File 4**).

A subsequent scRNA-seq analysis of eleven chemotherapy treated primary, recurrent, and metastatic OSA tumor specimens (GSE152048) was conducted using the same canonical markers and cellular clusters. Again, while *CD320* was ubiquitously expressed across numerous cellular populations, *TCN2* expression was concentrated on Myeloid cells. Unfortunately, subsequent quantification of Plasmocyte and B cell clusters revealed minimal B lymphocyte infiltration in the chemotherapy treated OSA tumors examined. Interestingly, these OSA samples also had substantially lower overall *TCN2+* Myeloid cell populations in comparison to the previously analyzed naive primary tumors (GSE162454). Additionally, the percentage (%) of *TCN2*+ Myeloid cells (relative to all Myeloid cells) was no greater than 21.26% across all tumors examined (**Supplementary File 1** – **Supplementary Figure 7**, **Supplementary File 4**). Overall, these analyses supported that *TCN2*+ Myeloid and *CD320+* Plasmocyte and B cell clusters were ultimately present in OSA tumors. Considering the previous publication’s note regarding isolation of these B lymphocytes from confirmed TLSs in one naive primary tumor (72), we also associated the highest % of TCN2+ Myeloid cells (relative to all Myeloid cells) with intratumoral TLS formation. Ultimately, investigation of additional OSA tumor specimens (positive for B lymphocyte and/or Plasmocyte populations) and further cell-cell communication analysis is required to support the hypothesis that *TCN2*+ Myeloid cells drive infiltration and proliferation of B lymphocyte populations through increased TCN2 (ligand) and CD320 (receptor) interactions.

### 3.8 Proteomic correlation analysis of APO-TCN2 reveals detectable signatures associated with plasma cell maintenance, B cell activation, TLS formation, and circulating immunoglobulins

**Figure 7:**
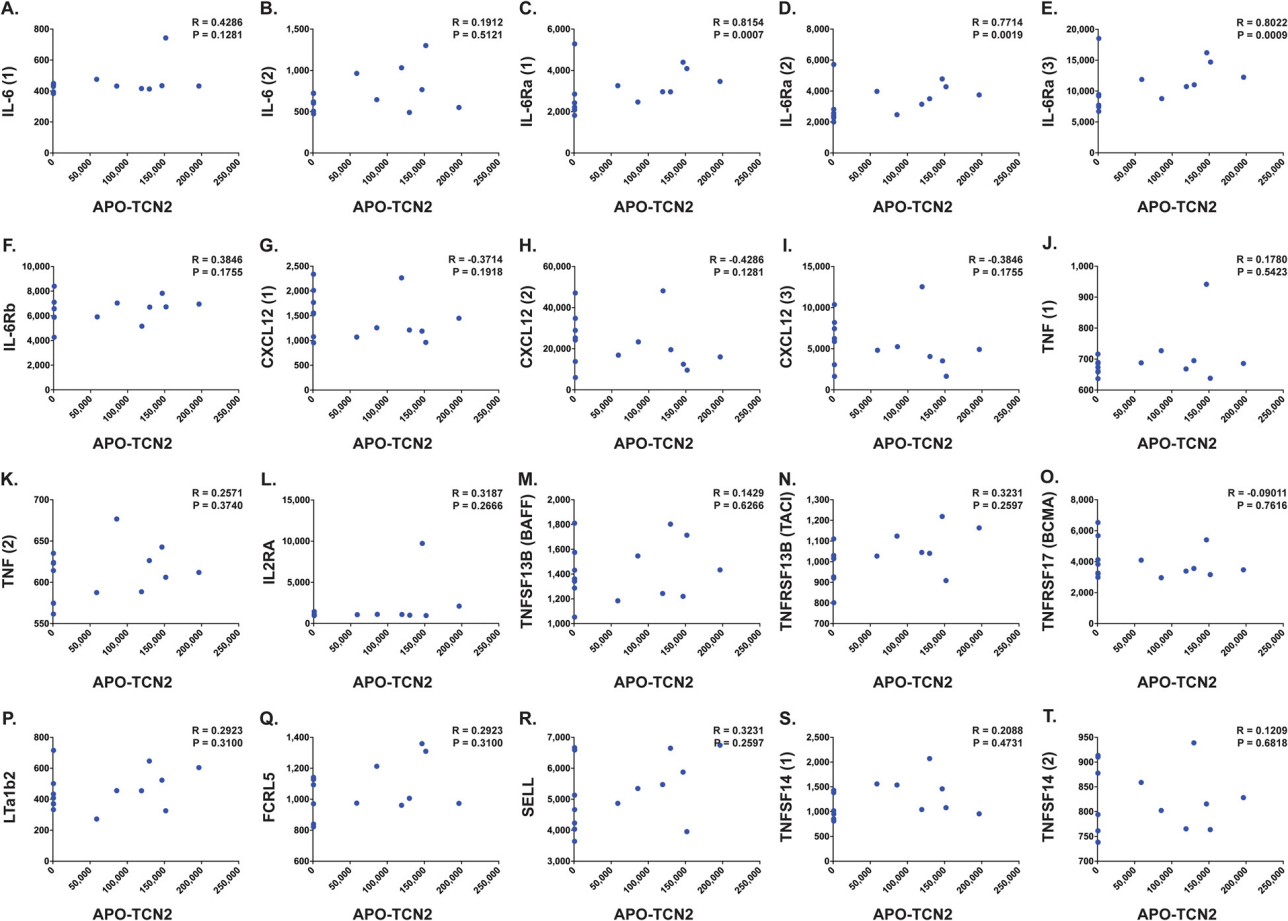
Correlation between APO-TCN2 and markers of plasma cell maintenance, B cell activation, and TLS formation. Spearman correlation analysis between measured APO-TCN2 (x-axis, RFUs) and markers of plasma cell maintenance including IL-6 [seq.2573.20, seq.4673.13] **(A, B)**, IL-6Ra [seq.15602.43, seq.4139.71, seq.8092.29] **(C–E)**, IL-6Rb [seq.2620.4] **(F)**, CXCL12 [seq.2330.2, seq.3516.60, seq.9278.9] **(G–I)**, and TNF [seq.5692.79, seq.5936.53] **(J, K)**, markers of B cell activation including IL2RA [seq.3151.6] **(L)**, TNFSF13B [seq.3059.50] **(M)**, TNFRSF13B [seq.2704.74] **(N)**, and TNFRSF17 [seq.2665.26] **(O)**, as well as markers of TLS formation including LTa1b2 [seq.3505.6] **(P)**, FCRL5 [seq.6103.70] **(Q)**, SELL [seq.4831.4] **(R)**, and TNFSF14 [seq.5355.69, seq.5988.49] **(S, T)** (y-axis, RFUs). Spearman correlation coefficient (R) and P-value (P) presented on each scatter plot. Each chemokine identified by multiple SOMAmers [example: TNFSF14 (1) and (2), representing seq.5355.69 and seq.5988.49, respectively] is presented with multiple correlation plots.

B lymphocyte infiltration, proliferation, and formation of TLSs is well regarded as a clinical biomarker of long term survival and response to immunotherapy in many solid tumors (92–115), including soft-tissue sarcoma (116, 117). Previous literature has suggested the necessity of the CD320 receptor for germinal center B cell growth and proliferation (83). Considering the apparent association between myeloid-derived TCN2 and CD320+ B lymphocytes in various solid tumors, a subsequent correlation analysis between plasma APO-TCN2 and previously described soluble markers of plasma cell maintenance (73), B cell activation (74), proteins critical for TLS formation (75), chemokines of the 12-CK signature (75) [exception – CCL4 (not measured)], and circulating immunoglobulin levels was then conducted. To begin, for those markers of plasma cell maintenance (73) (**Figures 7A–K**), positive correlations between plasma APO-TCN2 and IL-6 (**Figures 7A, B**), IL-6Ra (**Figures 7C–E**), IL-6Rb (**Figure 7F**), and TNF (**Figures 7J, K**) were observed, with statistical significance apparent for IL-6Ra. Additionally, a negative correlation between APO-TCN2 and CXCL12 (**Figures 7G–I**) was evident. Furthermore, for the soluble markers of B cell activation (74) (**Figures 7L–O**), IL-2RA (**Figure 7L**), TNFSF13B (BAFF) (**Figure 7M**), and TNFRSF13B (TACI) (**Figure 7N**) all displayed positive yet nonsignificant correlations with plasma APO-TCN2, while a negative, nonsignificant correlation was reported for TNFRSF17 (BCMA) (**Figure 7O**). Overall, these data suggest that increases in plasma APO-TCN2 were associated with increases in previously described soluble markers of B lymphocyte activity, albeit nonsignificant for most associations.

**Figure 8:**
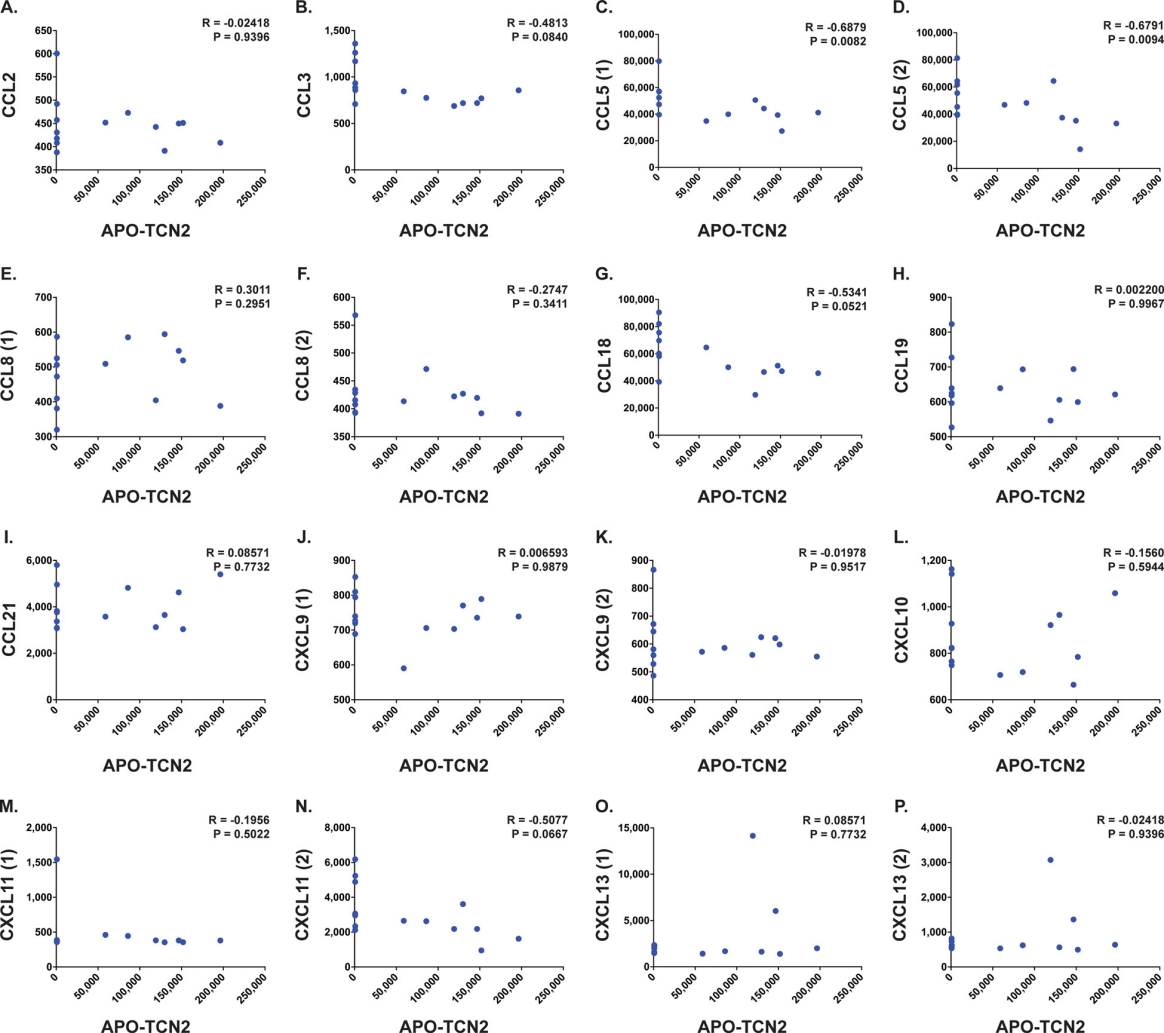
Correlation analysis between APO-TCN2 and measured chemokines of the 12-CK signature. Spearman correlation analysis between measured APO-TCN2 (x-axis, RFUs) and CCL2 [seq.2578.67] **(A)**, CCL3 [seq.3040.59] **(B)**, CCL5 [seq.2523.31, seq.5480.49] **(C, D)**, CCL8 [seq.13748.4, seq.2785.15] **(E, F)**, CCL18 [seq.3044.3] **(G)**, CCL19 [seq.4922.13] **(H)**, CCL21 [seq.2516.57] **(I)**, CXCL9 [seq.11593.21, seq.9188.119] **(J, K)**, CXCL10 [seq.4141.79] **(L)**, CXCL11 [seq.18171.25, seq.3038.9] **(M, N)**, CXCL13 [seq.13701.2, seq.3487.32] **(O, P)** (y-axis, RFUs) in naive OSA patient plasma samples (n = 14). Spearman correlation coefficient (R) and P-value (P) presented on each scatter plot. Each chemokine identified by multiple SOMAmers [example: CCL5 (1) and (2), representing seq.2523.31 and seq.5480.49, respectively] is presented with multiple correlation plots.

Furthermore, analysis of various proteins critical for TLS formation (75) (**Figures 7P–T**) revealed similar trends. Positive yet nonsignificant correlations between APO-TCN2 and LTa1b2 (**Figure 7P**), FCRL5 (**Figure 7Q**), SELL (**Figure 7R**), and TNFSF14 (**Figures 7S, T**) were observed, suggesting a possible association of APO-TCN2 levels with those proteins necessary for TLS formation. Furthermore, negative, nonsignificant correlations between APO-TCN2 and most chemokines of the 12-CK signature (75) were evident (**Figure 8**). Overall, these data [much like the findings for CXCL12 (**Figures 7G–I**)] may suggest that increased plasma APO-TCN2 is associated with the development of exacerbated chemokine axes (lower blood plasma and higher tissue levels) for potent lymphocytic infiltration into patient solid tumor and/or other tissues. Additional tissue/tumor-level analyses are necessary to further support this finding. Of note, additional correlation analyses of circulating immunoglobulin levels suggested negative correlations between plasma APO-TCN2 and IgA, IgD, IgE, IgM, and IgG, with a positive correlation to J chain (**Supplementary File 1** – **Supplementary Figure 8**). Limited research has correlated peripheral blood immunoglobulin levels with intratumoral TLSs, therefore, the significance of these findings is unknown. However, these data may indicate the presence of improved intratumoral humoral immune responses (increased J chain) in those patients with elevated APO-TCN2. Overall, these data suggested that elevated APO-TCN2 levels may be associated with a peripheral blood plasma signature necessary for robust B lymphocyte proliferation and infiltration in OSA tumors.

### 3.9 KM Plotter Immunotherapy suggests association of *TCN2/CD320* with response to immunotherapy and formation of intratumoral TLSs

**Figure 9:**
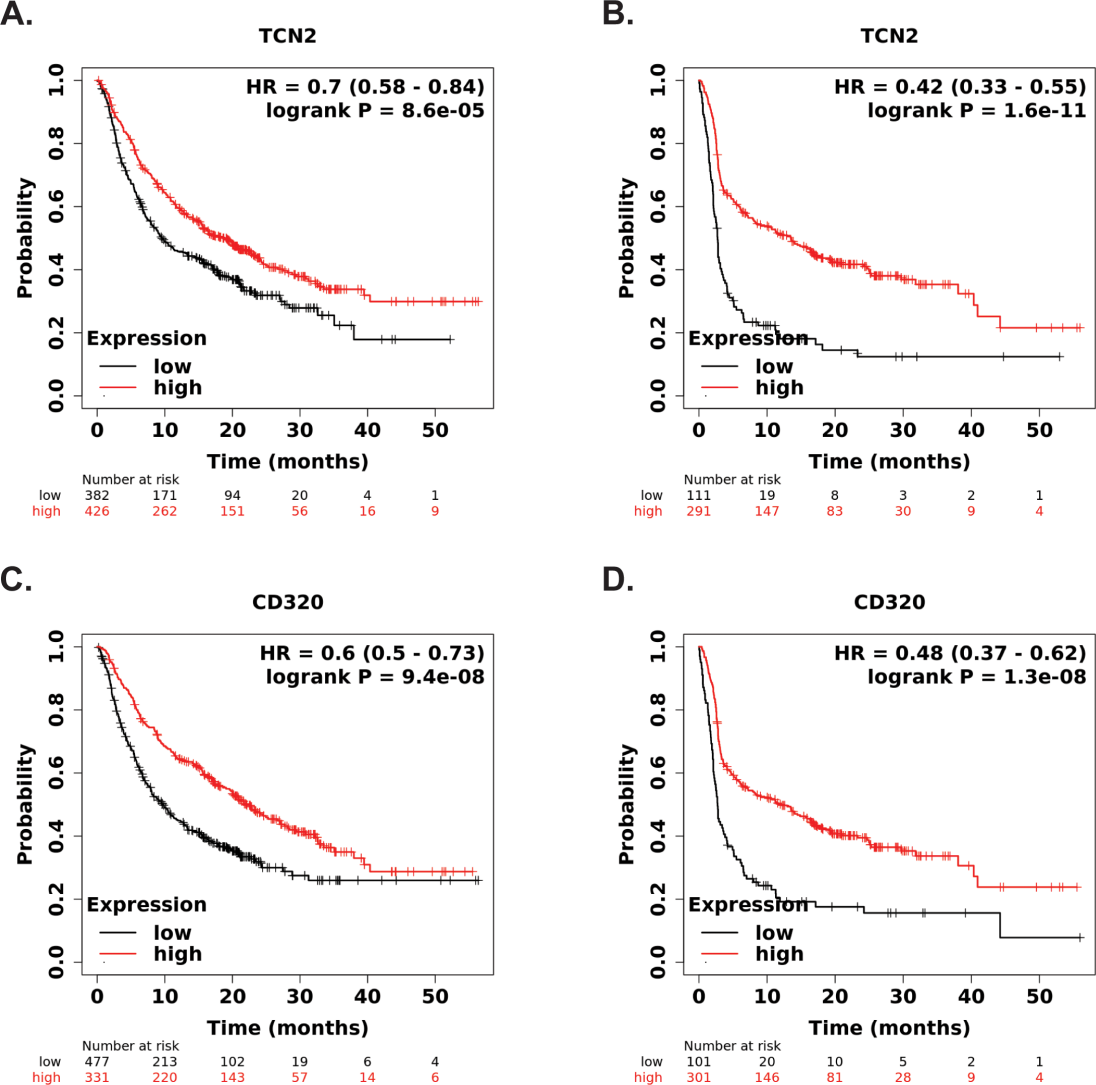
KM Plotter Immunotherapy survival analysis supports *TCN2* and *CD320* as a markers of immunotherapy response. Overall **(A)** and progression free **(B)** survival analysis of *TCN2*. Overall **(C)** and progression free **(D)** survival analysis of *CD320*. Each analysis included specimens from all solid tumors, collected pre-treatment, irrespective of immunotherapy target (anti-PD-1, anti-PD-L1, and anti-CTLA-4). Default KM Plotter settings were utilized with auto selection of best cutoff into high (red) and low (black) gene expression groups based on the calculation of all upper and lower quartiles with selection of the best performing threshold. KM survival curves with reported HR and log-rank P-value were constructed, with y-axis representing probability of survival and the x-axis representing time (months). The total number of patients at risk for each time point is reported. Survival curves generated using KM Plotter Immunotherapy.

Our group then hypothesized that if increases in plasma APO-TCN2 (and as a result, intratumor *TCN2*) is associated with lymphocytic infiltration and possible formation of TLSs, then increased *TCN2/CD320* expression in solid tumors, treated with immunotherapy, should correlate with better overall survival. To investigate this hypothesis, both *TCN2* and *CD320* were evaluated for overall and progression free survival in solid tumors treated with various immunotherapies (anti-PD-1, anti-PD-L1, anti-CTLA-4) using the KM Plotter Immunotherapy online database (76). Increased intratumoral expression of *TCN2* was indeed associated with better overall (log-rank P = 8.6e-05, FDR = 0.03) (**Figure 9A**) and progression free (log-rank P = 1.6e-11, FDR = 0.01) (**Figure 9B**) survival. Similar findings were also evident for the TCN2 receptor, as increased *CD320* expression was associated with better overall (log-rank P = 9.4e-08, FDR = 0.01) (**Figure 9C**) and progression free (log-rank P = 1.3e-08, FDR = 0.01) (**Figure 9D**) survival in the same dataset. These data suggest that increased pre-treatment *TCN2* and *CD320* expression is associated with better outcomes in a variety of solid tumors treated with immunotherapy.

To further examine whether intratumoral *TCN2* and *CD320* expression is associated with TLS formation, a correlation analysis to the 12-CK TLS signature (75) was performed using the KM Plotter Immunotherapy database (76). Unsurprisingly, increased expression of all 12-CK signature chemokines was significantly (FDR < 0.05) associated with better overall survival in the solid tumors examined (exception – CCL18, HR = 0.71, log-rank P = 0.00055, FDR = 0.20) (**Supplementary File 1** – **Supplementary Figure 9**). A Spearman correlation analysis supported significant (P < 0.05) positive correlations between *TCN2* and *CD320* expression with each chemokine of the 12-CK TLS signature (exception – *CD320* and *CCL4*) (**Supplementary File 1** – **Supplementary Table 3**). A subsequent multi-gene Spearman correlation analysis between *TCN2* and *CD320* with the 12-CK TLS signature (75) using the GEPIA2 web server and solid tumors of the TCGA database (77) was also performed. Importantly, *TCN2* expression displayed a significant positive correlation to the 12-CK gene signature for TCGA-SARC (P-value = 5.1e-13, R = 0.43). Similar results were evident for a combinatory analysis of all TCGA tumors (P-value = 3.4e-123, R = 0.24). In contrast, *CD320* expression displayed an opposite trend, with a weak negative correlation in both datasets (**Supplementary File 1** – **Supplementary Figure 10**). Overall, these data highlight a positive correlation between *TCN2* and the 12-CK TLS signature in a variety of solid tumors. While preliminary, these data may suggest an association between elevated peripheral plasma APO-TCN2 (alongside probable increases in intratumoral *TCN2* expression) with formation of TLSs. Further investigation of sarcoma tumor specimens is ultimately necessary to associate increased plasma APO-TCN2 with intratumoral B lymphocyte aggregation and/or TLS formation.

## 4 Discussion

This proteomic biodiscovery analysis of OSA patient plasma identified apo-transcobalamin-II (APO-TCN2) as a novel circulatory biomarker of survival in this disease. To our knowledge, the only known publication which previously characterized TCN2 in OSA was published by Rothzerg et al. in 2021. Here, *TCN2* was identified as one of four upregulated genes associated with better overall survival in OSA tumors of the TARGET-OS dataset (118). To our knowledge, no further characterization of TCN2 in OSA has since been conducted. Ultimately, the work presented here provides the utmost support for its relevance in this disease, as an over 6-fold increase in circulating APO-TCN2 levels was associated with better overall survival in our patient cohort. The dual association of both increased *TCN2* expression (in OSA tumors) and increased circulatory APO-TCN2 levels (in OSA peripheral blood plasma) with improved patient outcome provides credence to our findings and this biomarker’s future clinical utility in this disease. While additional studies to correlate intratumoral *TCN2* expression with plasma APO-TCN2 levels are necessary, these data suggest that circulating protein measurements (through liquid biopsy) could act as a surrogate for tumoral assessments in both local and advanced OSA. Future studies to assess changes in peripheral APO-TCN2 throughout the course of disease through serial blood sampling could also further associate plasma protein levels with disease progression, therapeutic response, and/or clinical remission.

TCN2’s biological function of supporting cellular metabolic processes by transporting vitamin B12 (cobalamin, co-factor for various enzymes) through the blood stream, binding to the ubiquitously expressed CD320 surface receptor, and internalization of the receptor-ligand complex for B12’s intracellular release is well known (119–121). Interestingly, numerous reports of TCN2 deficiency due to loss-of-function mutations have been described. In addition to the common presentation of failure to thrive and megaloblastic anemia (due to elevation of homocysteine and methylmalonic acid), a number of these cases reported immunological deficiencies and abnormal immunity including measured pancytopenia, neutropenia, and hypogammaglobulinemia (78–81, 122–138). TCN2 deficiency was also previously associated with abnormal granulocyte function and limited antimicrobial response to *Staphylococcus aureus* infection (139). Of note, no difference in HOLO-TCN2 levels were measured between our 2-year deceased and survivor cohorts, ruling out the possibility of a genetic abnormality in those deceased patients. These reports, however, highlight TCN2’s integral role in proliferation and maintenance of cells of the immune system.

In this regard, increased TCN2 levels were previously reported in patients with a variety of inflammatory and lymphoproliferative disorders, including those diagnosed with multiple myeloma and Waldenstrom macroglobulinemia experiencing overproduction of immunoglobulins (hyperglobulinemia) (140). McLean et al. later determined that TCN2 could not only drive proliferation of human erythroleukemic and murine lymphoma cell lines *in vitro*, but antibody blockade of its receptor could also effectively inhibit cellular growth (89, 90). Furthermore, TCN2 levels in peripheral blood monocytes of patients with various inflammatory bowel diseases including shigellosis, ulcerative colitis, and Crohn’s were 3-4 times higher than those measured in healthy normal patients, with levels decreasing in the setting of clinical improvement (141). TCN2 was also elevated in the setting of various infectious diseases including malaria and typhus (142–144), supporting its role as a possible acute phase reactant (145).

Of utmost importance, increases in unsaturated TCN2 (APO-TCN2) have been reported in patients with active autoimmune diseases including systemic lupus erythematous (SLE), autoimmune hemolytic anemia, and dermatomyositis, with levels often correlating with disease activity (146–148). Haynes et al. in 2020 also identified a 93-gene signature for diagnosis of SLE through transcriptomic profiling of patient peripheral blood, of which *TCN2* was included (149). Additionally, while total serum cobalamin was no different between patients with active rheumatoid arthritis (RA) and clinical remission, APO-TCN2 was significantly elevated in those with active disease (150). These data suggest that APO-TCN2 is elevated in the setting of pathogenic immune responses in various immune-mediated disorders, often correlating with disease activity. Considering these clinical observations, Liu et al. recently investigated TCN2’s pathogenic role in a murine model of lupus. Increased expression of *TCN2* in both B and T lymphocytes of SLE patients and lupus-like mice were observed. Importantly, global genetic knockout of *TCN2* in this murine model of lupus resulted in complete amelioration of lupus symptoms, as measured by lower dsDNA levels, reduced IgG deposition in the glomerulus, and decreased infiltration of both B and T follicular helper cells in the kidneys and spleen of these mice. The group suggested that TCN2 activity is associated with the abnormal germinal center responses in the setting of SLE and could act as a novel therapeutic target in this disease (91). Considering these findings, it appears that the elevated APO-TCN2 in our 2-year survival cohort, with no difference in HOLO-TCN2 levels, mirrors the heightened systemic immune activity seen in common autoimmune disorders. These *in vivo* results suggest the importance of TCN2 for germinal center-mediated immune activity, as seen in the kidneys of patients with lupus nephritis (151). While the majority of previous literature indicated that TCN2 was merely correlative in nature (152), these data ultimately suggest a causative role for this circulating protein in the development and progression of lymphoproliferative autoimmune disease.

To suggest a possible mechanism by which APO-TCN2 improves overall survival in solid tumors, various analyses were then conducted using publicly available scRNA-seq datasets. Analysis of the TIGER database supported previous literature which characterized *TCN2* expression and active production from, among others, myeloid lineage (82, 153, 154) and endothelial (155, 156) cells. This analysis also confirmed the expression of *CD320* ubiquitously across many cell types (84) including B lymphocytes (86). The cell-cell communication analyses highlighted the interaction between B lymphocytes (positive for *CD320)* with Myeloid cells (positive for *TCN2*) in both NSCLC and SKCM. These results suggest the presence of a coordinated communication network between these cellular populations which likely supports intratumoral B lymphocyte proliferation for enhanced anti-tumor immune responses. Considering the significant correlation between *TCN2* and *CD320* with the 12-CK Score (75) and previous literature’s characterization of the CD320 receptor as necessary for germinal center B cell growth (83), these interactions may help coordinate the formation of intratumoral TLSs in these patients. With macrophages having been previously described as potent lymphoid tissue inducer cells necessary for T and B cell recruitment in these structures (157), and knockout of *TCN2* by Liu et al. inhibiting infiltration of B/T lymphocytes in kidneys and spleen in a murine model of lupus (91), further investigation of this intratumoral signaling network is therefore warranted.

Importantly, those B lymphocytes of the TIGER database with the greatest increased expression of *CD320* included *MZB1+* and immunoglobulin producing B cell clusters, commonly found in the marginal zone of germinal centers and tumor TLSs (158–160). Of the receptor-ligand interactions driving the cell-cell communication effect scores between these *TCN2+* Myeloid and *CD320+* B cell clusters, many are associated with promoting B cell activity. Of note, interactions between CD74 and MIF have been previously associated with both peripheral B cell survival (161) and chemotaxis (162, 163). Additionally, while interactions between CD74 and COPA (COPI coat complex subunit alpha) of malignant plasma and immune cells have been described in Waldenstrom macroglobulinemia (164), these interactions have also been shown to play a role in the maturation of B cells in the setting of antibody-mediated renal transplant rejection (165). Furthermore, scRNA-seq analysis of peripheral immune cells in patients with primary Sjogren’s syndrome highlighted not only enhanced CD74-COPA interactions between monocytes and naive B cells, but also CD74-amyloid beta precursor protein (APP) interactions between monocytes and memory B cells. Considering their enrichment in those with active disease, these receptor-ligand interactions were thought to be important drivers of autoimmunity (166). Further receptor-ligand interactions between HLA class II histocompatibility antigen, DP beta chain (HLA-DPB1) and TNFSF13B (BAFF) in addition to Major Histocompatibility Complex, Class II, DP Alpha 1 (HLA-DPA1) and TNF Superfamily Member 9 (TNFSF9) suggest myeloid-mediated B lymphocyte survival and maturation (167) in germinal centers of these solid tumors, as previously proposed (168). The association of high *TCN2* and *CD320* expression with better overall survival in patients treated with immunotherapy could likely reflect the increased propensity for B lymphocyte survival, proliferation, and maturation for TLS formation within the responding patient tumors (107).

While only preliminary, both *TCN2+* Myeloid and *CD320+* Plasmocytes and B cells were identified by scRNA-seq analyses of OSA tumor specimens. Most B lymphocytes characterized in the original analysis were confirmed to have been isolated from a single primary tumor containing TLSs (72). The identification of intratumoral B cells associated with TLSs in one of six naive tumors reflects the rates previously described in the amended PEMBROSARC trial of STS (approx. 20% of patients on initial screening) (169). These preliminary results, however, suggest the necessity for elevated *TCN2*+ Myeloid cells for B lymphocyte proliferation in the OSA tumor microenvironment. Additional analyses of known TLS-positive and -negative OSA or sarcoma tumor specimens are necessary to further support this interactive mechanism in sarcomas disease. Importantly, various correlation analyses presented here may indicate that elevated APO-TCN2 was associated with a favorable B lymphocyte (73, 74) proteomic profile likely necessary for potent immune cell recruitment and TLS-formation (75) to drive antibody-mediated anti-tumor immune responses in the OSA patients examined. Ultimately, while the mechanism by which *TCN2* is associated with better overall survival in OSA was extensively postulated in these analyses, further confirmatory studies are indeed necessary.

Of note, a distinct biological function of APO-TCN2 has, to our knowledge, not yet been described. The specific elevation of APO-TCN2 (as opposed to HOLO-TCN2) was previously associated with various lymphoproliferative autoimmune disorders but not further investigated. Our group, however, can postulate several mechanisms by which the increased circulatory APO-TCN2, measured here in OSA, is associated with better overall survival. First and foremost, our group hypothesizes that increased circulatory APO-TCN2 likely reflects the development of systemic and local proliferative immune responses, driven by activated myeloid lineage cells (82), as a result of immune system detection of cancer. Previous literature suggests that not only do activated lymphocytes have a preference for TCN2 over other carriers of cobalamin (170), but TCN2 is also preferentially absorbed by normal tissues and organs, even in the setting of proliferative tumorigenesis (171). More than likely, increased APO-TCN2 improves the probability of delivering vitamin B12 (through HOLO-TCN2) to lymphoid organs to drive robust immune proliferation and activated immune responses. The elevated APO-TCN2 levels within the survivor patient cohort would then reflect an increased demand for cobalamin in the setting of enhanced lymphocytic proliferation. We provided evidence that this proliferation is occurring within B lymphocytes and associated with intratumoral germinal center formation for enhanced anti-tumor immune responses.

Secondarily, previous literature suggests that APO-TCN2 can likely act as a competitive inhibitor of the CD320 receptor. In this scenario, APO-TCN2 could block the binding and delivery of vitamin B12 (HOLO-TCN2) on CD320+ cells (172), such as malignant tumor cells, thereby limiting their proliferation. While possible, our group believes APO-TCN2’s anti-tumor effect is more than likely through direct lymphocyte activation and proliferation as opposed to activity on tumor cells as a competitive inhibitor. Additionally, APO-TCN2 could possibly drive an undescribed signaling mechanism in these cells, associated with immune activation or pro-inflammatory signal transduction, through binding the CD320 receptor, binding the previously described megalin [thought to be essential in the accumulation of TCN2 in the kidney cortex and other absorptive epithelia (173)], binding an undefined receptor, or forming an additional receptor-ligand complex. Efforts to characterize the activity (if any) of APO-TCN2 are necessary for better understanding its role in both OSA and autoimmune disease. Nevertheless, these results suggest that surviving OSA patients are mounting a robust, systemic immune response as reflected through elevated levels of a biomarker (APO-TCN2) previously associated with active lymphoproliferative autoimmune disease.

While robust in our analyses, this study has inherent limitations. To begin, our plasma proteomic investigation analyzed only 14 naive OSA patients, with n = 3 in the 2-year deceased and n = 11 in the 2-year survival cohort. Considering the limited number of patient samples and the exploratory nature of the study, DEPs between comparative groups were determined based on having a Log2FC > 0.585 or < -0.585 and P-value < 0.05, leading to the possible discovery of false positive DEPs. Future investigations with larger sample sizes will utilize more stringent statistical measures [false discovery rate (FDR), corrected P-values (Bonferroni)] to determine DEPs. Importantly, however, the major finding from this study was supported by gene expression analysis of the largest known OSA RNA-seq dataset (TARGET-OS) in addition to other solid tumors (i.e. TCGA-SARC). Furthermore, several of the analyses presented here, including APO-TCN2’s relationship to other plasma proteins and scRNA-seq cell-cell communication analysis, are mostly correlative and associative in nature. These findings ultimately require further validation in confirmatory studies. Additionally, as to be expected with clinical specimens, the Streck-collected plasma samples analyzed here were processed and stored over varying periods of time. It is well understood that delays in sample processing can affect the measured proteome of patient samples. Fortunately, a previous analysis by Savage et al. using the O-link proteomic platform reported no change in TCN2 levels (in comparison to fresh samples) even after 18 hours of room temperature storage prior to blood specimen processing (log2FC = -0.0087, P-value = 0.860127) (174). These results suggest that the measured APO-TCN2 levels reported here were likely minimally influenced by differential processing times. Ultimately, future studies which plan to expand our OSA patient cohort will be more cognizant of sample collection and processing time to limit confounding influences during comparative analyses.

In conclusion, this study identified apo-transcobalamin-II (APO-TCN2) as a novel plasma proteomic biomarker of survival in OSA. We provide evidence that increases in APO-TCN2 likely reflect a systemic and local inflammatory myeloid response which drives proliferation and intratumoral infiltration of B lymphocytes for improved anti-tumor immunity in these patients. This finding correlated with the only known documented investigation of TCN2 in this disease, which previously suggested *TCN2* as one of four upregulated survival genes in OSA tumors of the TARGET-OS dataset (118). Further expansion of our patient cohort for validation of APO-TCN2’s clinical utility as a biomarker of improved overall survival is warranted. Future studies to investigate the distinct biological function of APO-TCN2 in both OSA and lymphoproliferative autoimmune disorders are necessary. Considering the availability of recombinant forms of this globulin protein, studies which investigate therapeutic injection of APO-TCN2 in murine models of metastatic OSA could ultimately suggest a future therapeutic role for this protein. Exogenous TCN2 may be critical to drive the robust expansion and lymphocytic proliferation necessary for enhanced anti-tumor immune responses in these patients.

## Data Availability

The datasets used and/or analyzed during this study are available in the Supplementary Files, the GEO repository (https://www.ncbi.nlm.nih.gov/geo/query/acc.cgi?acc=GSE162454, https://www.ncbi.nlm.nih.gov/geo/query/acc.cgi?acc=GSE152048), the corresponding web-based platforms (https://bhasinlab.bmi.emory.edu/Survival Genie/, http://tiger.canceromics.org/#/, https://kmplot.com/analysis/, http://gepia2.cancer-pku.cn/#index), or from the corresponding author on reasonable request.

## Supplementary material

Supplementary File 1 Supplementary Figures and Tables: **Supplementary Figures 1**-**11** and **Supplementary Tables 1**-**3** including titles and legends.

Supplementary File 2 All Naive OSA, 2-year Survival Analysis: Raw proteomics data (RFUs), comparison between naive, all OSA, 2-year survival analysis, and Reactome pathway analysis.

Supplementary File 3 Naive, Advanced Disease OSA, 2-year Survival Analysis: Raw data for comparison between naive, advanced disease OSA, 2-year survival analysis with additional data from Reactome pathway analysis.

Supplementary File 4 scRNA-seq of Naive and Chemotherapy Treated OSA tumor: Raw data derived from Loupe Browser used in analysis of various cellular populations of interest and reported in manuscript.

## Glossary

12-CK: 12-chemokine
ACP5: Acid Phosphatase 5, Tartrate Resistant
ACTA2: Actin Alpha 2, Smooth Muscle
AHSG: Alpha 2-HS Glycoprotein
ALPL: Alkaline Phosphatase, Biomineralization Associated
ANG: Angiogenin
APO-TCN2: Apo-Transcobalamin-II
APP: Amyloid Beta Precursor Protein
AT2: Alveolar epithelial type II
BC: Breast Cancer
C1QA: Complement C1q A Chain
C5AR1: Complement C5a Receptor 1
CAT: Catalase
CCL18: C-C Motif Chemokine Ligand 18
CCL19: C-C Motif Chemokine Ligand 19
CCL2: C-C Motif Chemokine Ligand 2
CCL21: C-C Motif Chemokine Ligand 21
CCL3: C-C Motif Chemokine Ligand 3
CCL4: C-C Motif Chemokine Ligand 4
CCL5: C-C Motif Chemokine Ligand 5
CCL8: C-C Motif Chemokine Ligand 8
CCR1: C-C Motif Chemokine Receptor 1
CD2: T-Cell Surface Antigen CD2
CD320: Transcobalamin Receptor
CD3D: CD3 Delta Subunit Of T-Cell Receptor Complex
CD3E: CD3 Epsilon Subunit Of T-Cell Receptor Complex
CD3G: CD3 Gamma Subunit Of T-Cell Receptor Complex
CD44: Cluster of differentiation 44
CD68: Cluster of differentiation 68
CD74: Cluster of differentiation 74
CD79A: Cluster of differentiation 79A
CD94: Cluster of differentiation 94
CKB: Creatine Kinase B
CKM: Creatine Kinase, M-Type
COL1A1: Collagen Type I Alpha 1 Chain
COPA: COPI Coat Complex Subunit Alpha
CP: Cutpoint
CRC: Colorectal cancer
CTC: Circulating tumor cells
ctDNA: Circulating tumor DNA
CTLA-4: Cytotoxic T-Lymphocyte Associated Protein 4
CTSK: Cathepsin K
CXCL10: C-X-C Motif Chemokine Ligand 10
CXCL11: C-X-C Motif Chemokine Ligand 11
CXCL12: C-X-C Motif Chemokine Ligand 12
CXCL13: C-X-C Motif Chemokine Ligand 13
CXCL4: C-X-C Motif Chemokine Ligand 4
CXCL6: C-X-C Motif Chemokine Ligand 6
CXCL9: C-X-C Motif Chemokine Ligand 9
DEP: Differential expressed protein
EDTA: Ethylenediaminetetraacetic acid
EGFL7: EGF Like Domain Multiple 7
FAM3C: FAM3 Metabolism Regulating Signaling Molecule C
FBP1: Fructose-Bisphosphatase 1
FCRL5: Fc Receptor Like 5
FDR: False discovery rate
FOXO: Forkhead Box-O
FPKM: Fragments Per Kilobase of transcript per Million mapped reads
GEPIA2: Gene Expression Profiling Interactive Analysis 2
GNLY: Granulysin
GRN: Granulin Precursor
GZMB: Granzyme B
HLA-DPA1: Major Histocompatibility Complex, Class II, DP Alpha 1
HLA-DPB1: Major Histocompatibility Complex, Class II, DP Beta 1
HLA-DRB1: HLA class II histocompatibility antigen, DRB1 beta chain
HLA-E: Major histocompatibility complex, class I, E
HOLO-TCN2: Holo-Transcobalamin-II
HR: Hazard Ratio
IBSP: Integrin Binding Sialoprotein
ICC: Intrahepatic Cholangiocarcinoma
IgA: Immunoglobulin A
IgD: Immunoglobulin D
IgE: Immunoglobulin E
IgG: Immunoglobulin G
IGHG1: Immunoglobulin Heavy Constant Gamma 1
IGJ: Joining Chain of Multimeric IgA and IgM
IgM: Immunoglobulin M
IL-6: Interleukin-6
IL-6Ra: Interleukin-6 receptor, alpha
IL-6Rb: Interleukin-6 receptor, beta
IL2RA: Interleukin-2 receptor subunit alpha
IRB: Institutional Review Board
KLRB1: Killer Cell Lectin Like Receptor B1
KLRD1: Killer Cell Lectin Like Receptor D1
KM: Kaplan Meier
LR: Log-rank
LTa1b2: Lymphotoxin alpha1:beta2
LYZ: Lysozyme
MAPK: Mitogen-activated protein kinases
MCC: Merkel cell carcinoma
MIF: Macrophage Migration Inhibitory Factor
MS: Mass Spectrometry
MS4A1: Membrane Spanning 4-Domains A1
MZB1: Marginal Zone B and B1 Cell Specific Protein
NCI: National Cancer Institute
TARGET-OS: Therapeutically Applicable Research to Generate Effective Treatments-Osteosarcoma
NKG2A: Killer Cell Lectin Like Receptor C1
NKG7: Natural Killer Cell Granule Protein 7
NPC: Nasopharyngeal carcinoma
NSCLC: Non-small cell lung carcinoma
OSA: Osteosarcoma
PD-1: Programmed cell death protein 1
PD-L1: Programmed death-ligand 1
PPA1: Inorganic Pyrophosphatase 1
RA: Rheumatoid Arthritis
RETN: Resistin
RFU: Relative fluorescent unit
RPS19: Ribosomal Protein S19
RUNX2: RUNX Family Transcription Factor 2
scRNA-seq: single cell RNA-sequencing
SELDI-TOF: Surface-enhanced laser desorption/ionization with time of flight
SELEX: Systemic Evolution of Ligands by Exponential
SELL: Selectin L
SKCM: Skin cutaneous melanoma
SLE: Systemic Lupus Erythematous
SPP1: Secreted Phosphoprotein 1
STAD: Stomach adenocarcinoma
t-SNE: t-distributed Stochastic Neighbor Embedding
TCGA: The Cancer Genome Atlas
SARC: Sarcoma
TCN2: Transcobalamin-II
TIGER: Tumor Immunotherapy Gene Expression Resource
TIL: Tumor-infiltrating lymphocyte
TIM-3 (HAVCR2): T-Cell Immunoglobulin and Mucin Domain-Containing Protein 3 (Hepatitis A Virus Cellular Receptor 2)
TLS: Tertiary lymphoid structure
TNF: Tumor necrosis factor
TNFRSF13B (TACI): TNF Receptor Superfamily Member 13B (Transmembrane Activator and CAML Interactor)
TNFRSF17 (BCMA): TNF Receptor Superfamily Member 17 (B-cell maturation antigen)
TNFRSF1A: TNF Receptor Superfamily Member 1A
TNFSF13B (BAFF): TNF Superfamily Member 13b (B-cell activating factor)
TNFSF14: TNF Superfamily Member 14
TNFSF9: TNF Superfamily Member 9
UMAP: Uniform Manifold Approximation and Projection
UMI: Unique Molecular Identifier
VEGF: Vascular endothelial growth factor
VIM: Vimentin
