# Supplementary material for: Proteomic and transcriptomic analyses identify apo-transcobalamin-II as a biomarker of overall survival in osteosarcoma
Source: Front Oncol. 2024 Oct 18;14:1417459. doi: 10.3389/fonc.2024.1417459 (PMC11527601; doi:10.3389/fonc.2024.1417459)
Supplement: Supplementary File 1 — Supplementary Figures and Tables: Supplementary Figures 1 - 11 and Supplementary Tables 1 - 3 including titles and legends. [file DataSheet1.docx]

**
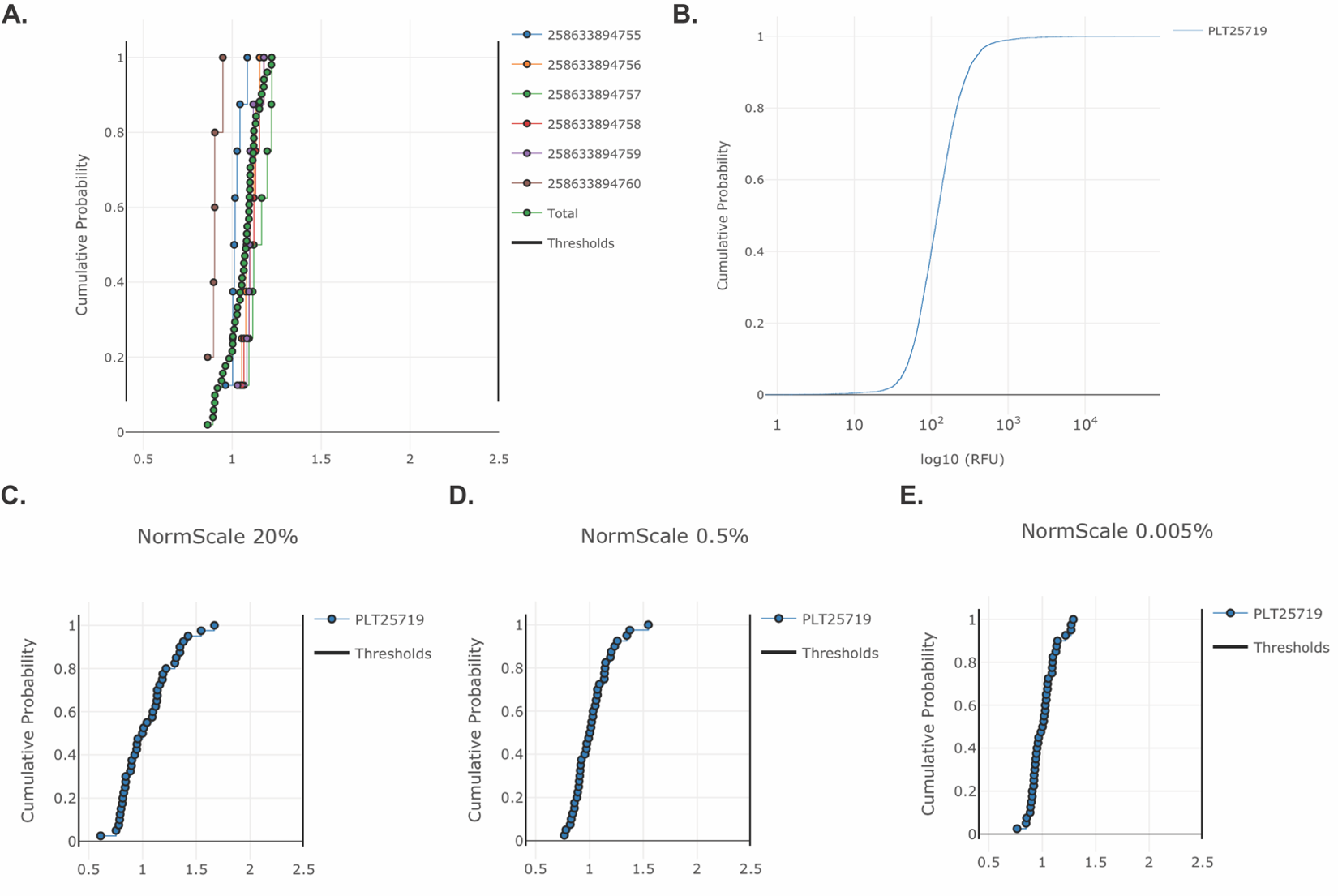
**

**Supplementary Figure 1 – SomaLogic QA/QC.** (A) Hybridization Scale Factors for normalization are within the expected range of 0.4 – 2.5. (B) Estimated Limit of Detection (eLOD) approximates the lowest protein signal reliably detected for each analyte measured. (C) Median Normalization Scale Factors for the 20% dilution group with values in expected range of 0.4 – 2.5. (D) Median Normalization Scale Factors for the 0.5% dilution group with values in expected range of 0.4 – 2.5. (E) Median Normalization Scale Factors for the 0.005% dilution group with values in expected range of 0.4 – 2.5.

**
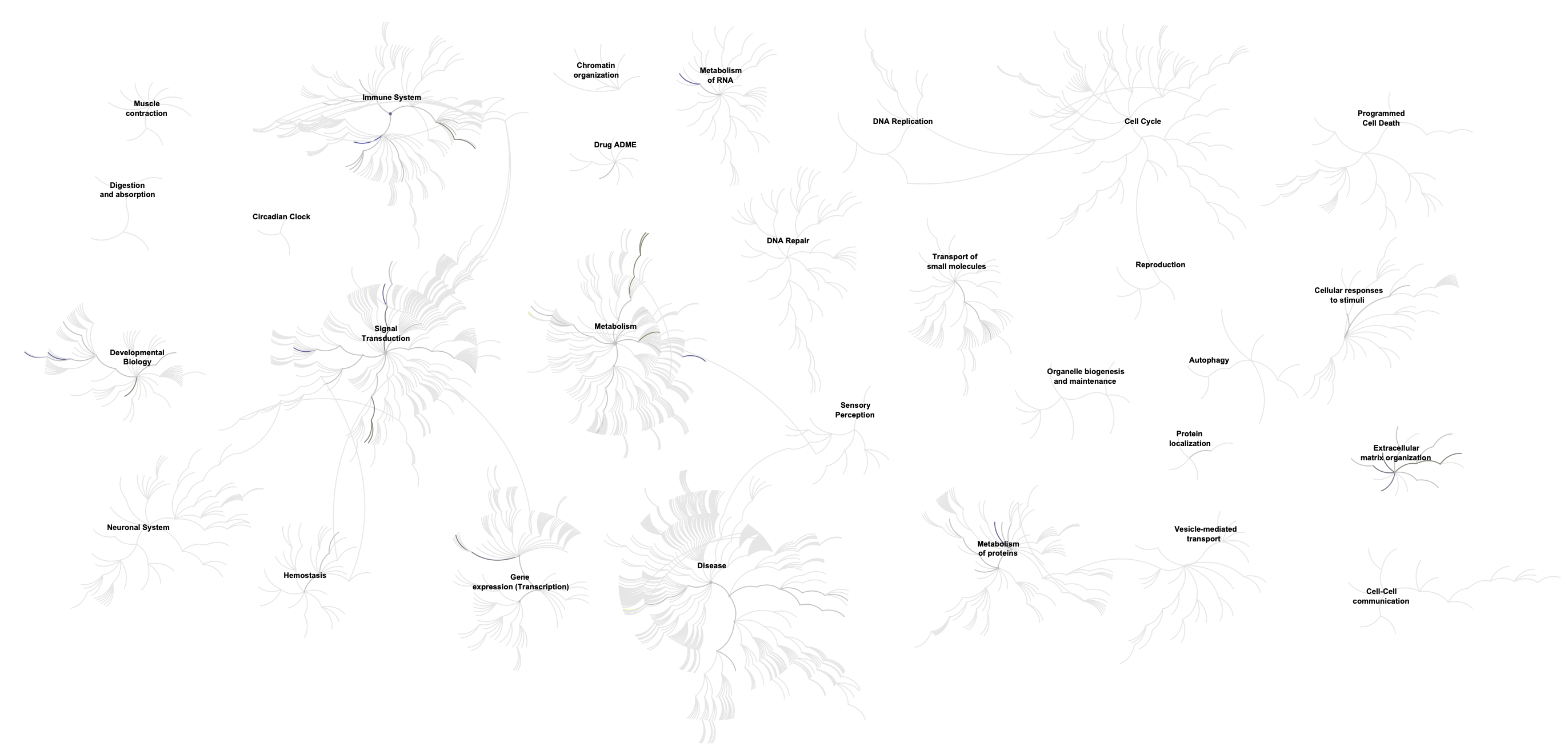
**

**Supplementary Figure 2 – Reactome genome wide overview map for all naïve OSA patients (n = 14), stratified by 2-year survival status.** Map highlights overrepresentation of pathways of the Immune System, Signal Transduction, and Metabolism for the DEPs of the 2-year deceased and survivor comparison.

**
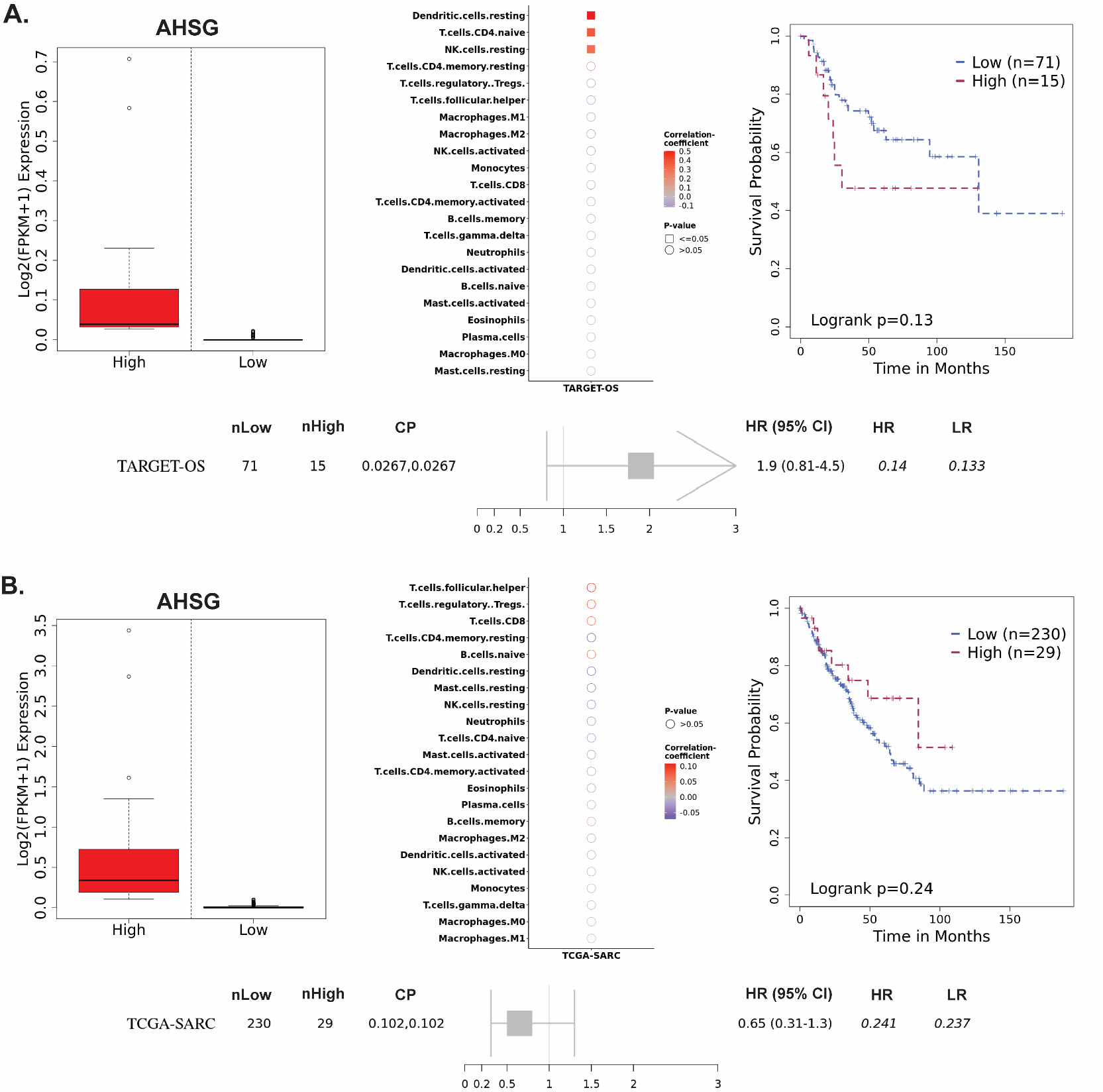
**

**Supplementary Figure 3 – TARGET-OS and TCGA-SARC survival analysis of AHSG.** Analysis of *AHSG* in TARGET-OS (A) and TCGA-SARC (B) databases using Survival Genie web-based platform. Box plots representing *AHSG* FPKM normalized expression in primary tumors with stratification into low and high expressing groups. The relative fraction of TILs was estimated using the tumor-infiltrating immune cell type matrix LM22 gene signature and CIBERSORTx deconvolution. Pearson correlation matrix of deconvoluted immune cell RNA-seq gene expression data and *AHSG*, with shape (square or circle) denoting significance and color denoting positive (red) or negative (blue) correlation with *AHSG*. Kaplan-Meier (KM) survival curves with high (red) and low (blue) group stratification are compared using the log-rank test, with log-rank P-value < 0.05 considered statistically significant. Forest plot details the association between the high and low groups [stratified by cut point (CP)] based on the Cox Proportional Hazards regression model. nLow and nHigh represent the number of patients in low and high expressing groups, respectively. Hazard ratio (HR) with 95% confidence interval as well as the associated wald-test (HR) and log-rank (LR) P-values are reported.

**
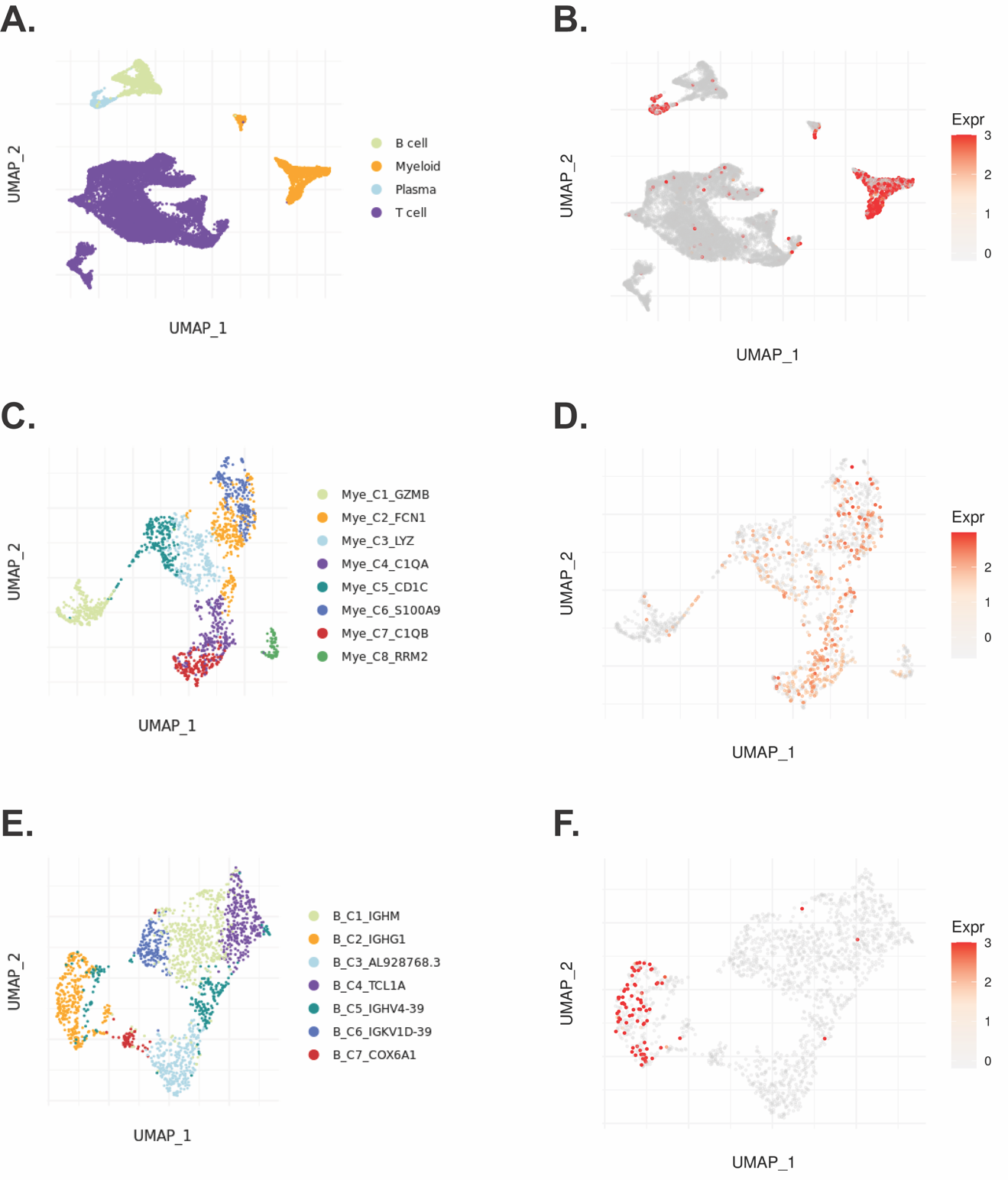
**

**Supplementary Figure 4 – TIGER database confirms *TCN2* expression on various myeloid and B cellular clusters of SKCM1 dataset.** (A-B) UMAPs of all cellular clusters of the SKCM1 dataset highlight expression of *TCN2* on myeloid cells. (C-D) UMAPs of the Myeloid subclusters of the SKCM1 dataset highlighting expression of *TCN2*. (E-F) UMAPs of the B cell subclusters of the SKCM1 dataset highlights expression of *TCN2*. UMAPs generated using the TIGER portal.

**
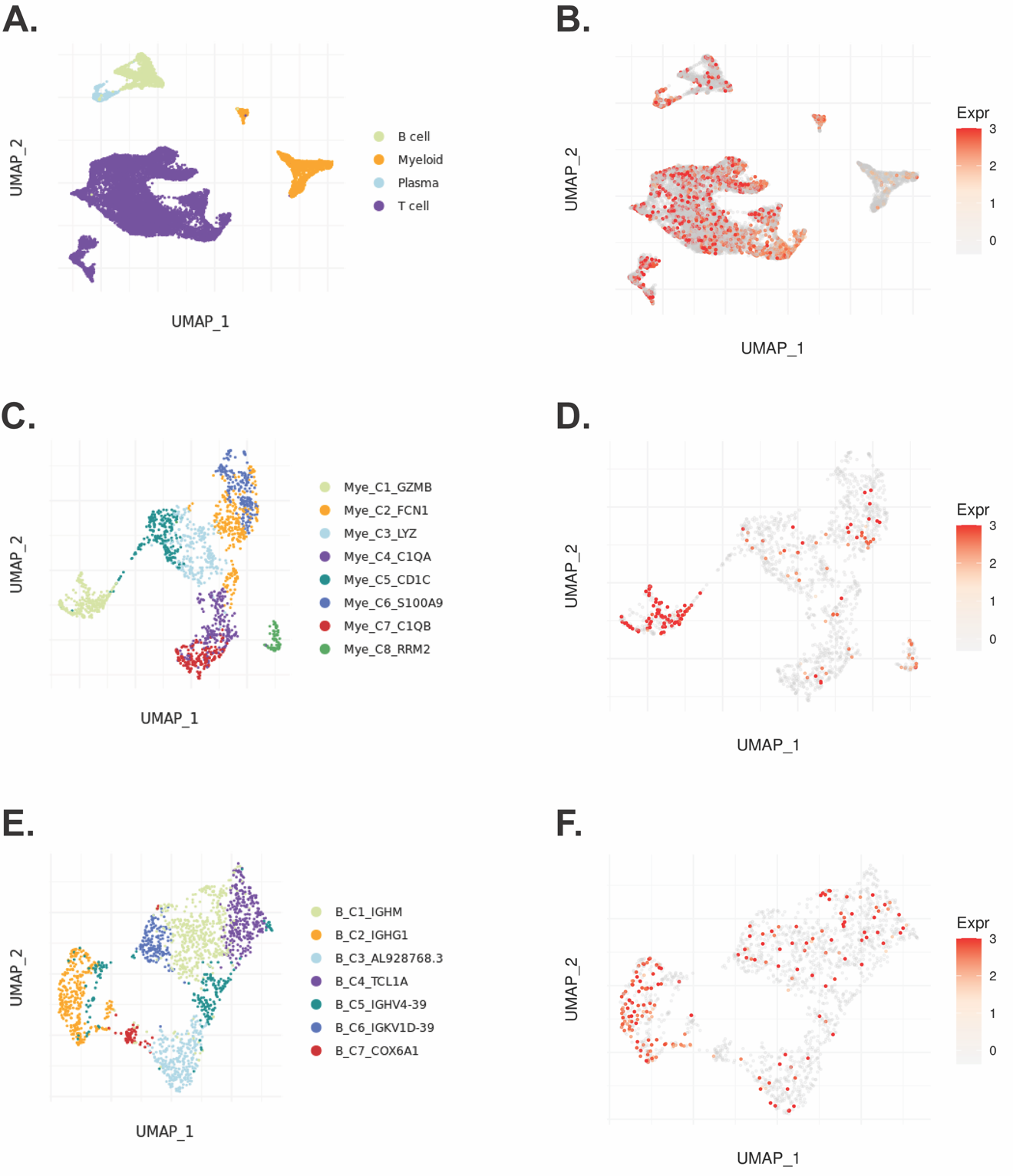
**

**Supplementary Figure 5 – TIGER database confirms *CD320* expression on various plasma, myeloid, and B cell clusters of SKCM1 dataset.** (A-B) UMAPs of all cellular clusters of the SKCM1 dataset highlight expression of *CD320* on various immune cells including plasma cells. (C-D) UMAPs of the Myeloid subclusters of the SKCM1 dataset highlighting expression of *CD320*. (E-F) UMAPs of the B cell subclusters of the SKCM1 dataset highlights expression of *CD320*. UMAPs generated using the TIGER portal.

**
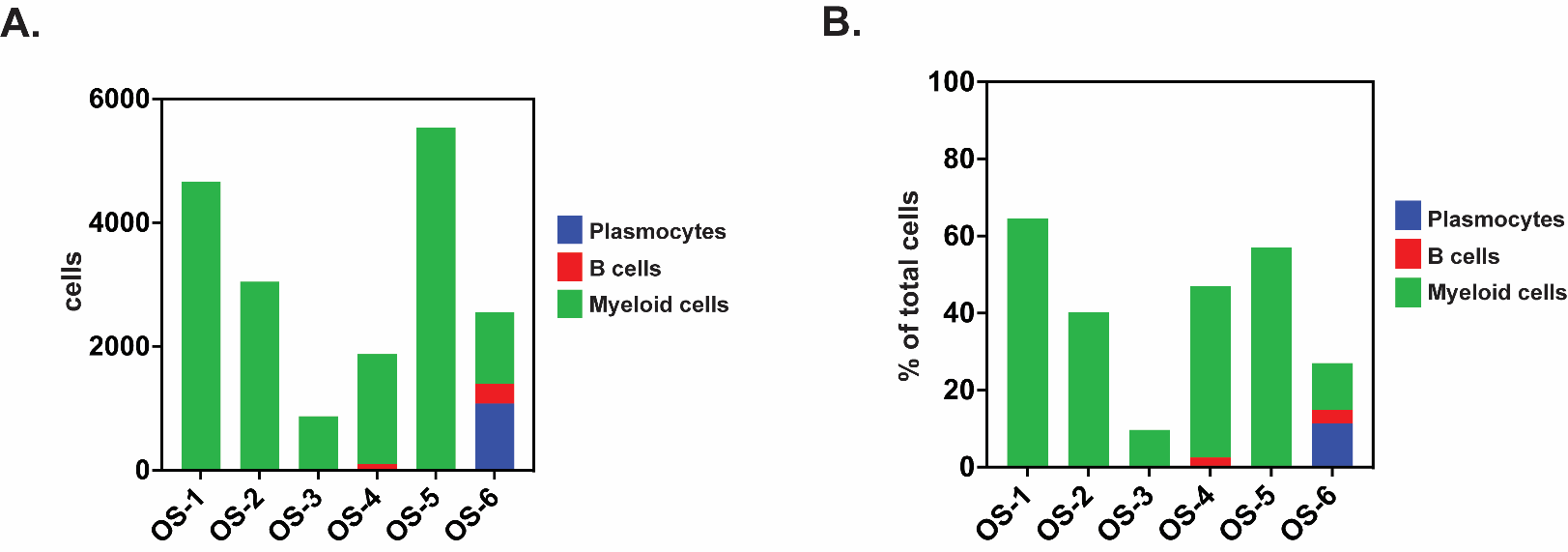
**

**Supplementary Figure 6 – Additional clustering data from naïve OSA primary tumors.** (A) Quantification of total cells for the Plasmocytes, B cells, and Myeloid cells populations in all naïve OSA patient tumors (n = 6). (B) Quantification of the percent (%) of total cells for Plasmocytes, B cells, and Myeloid cells population in all naïve OSA patient tumors (n = 6).

**
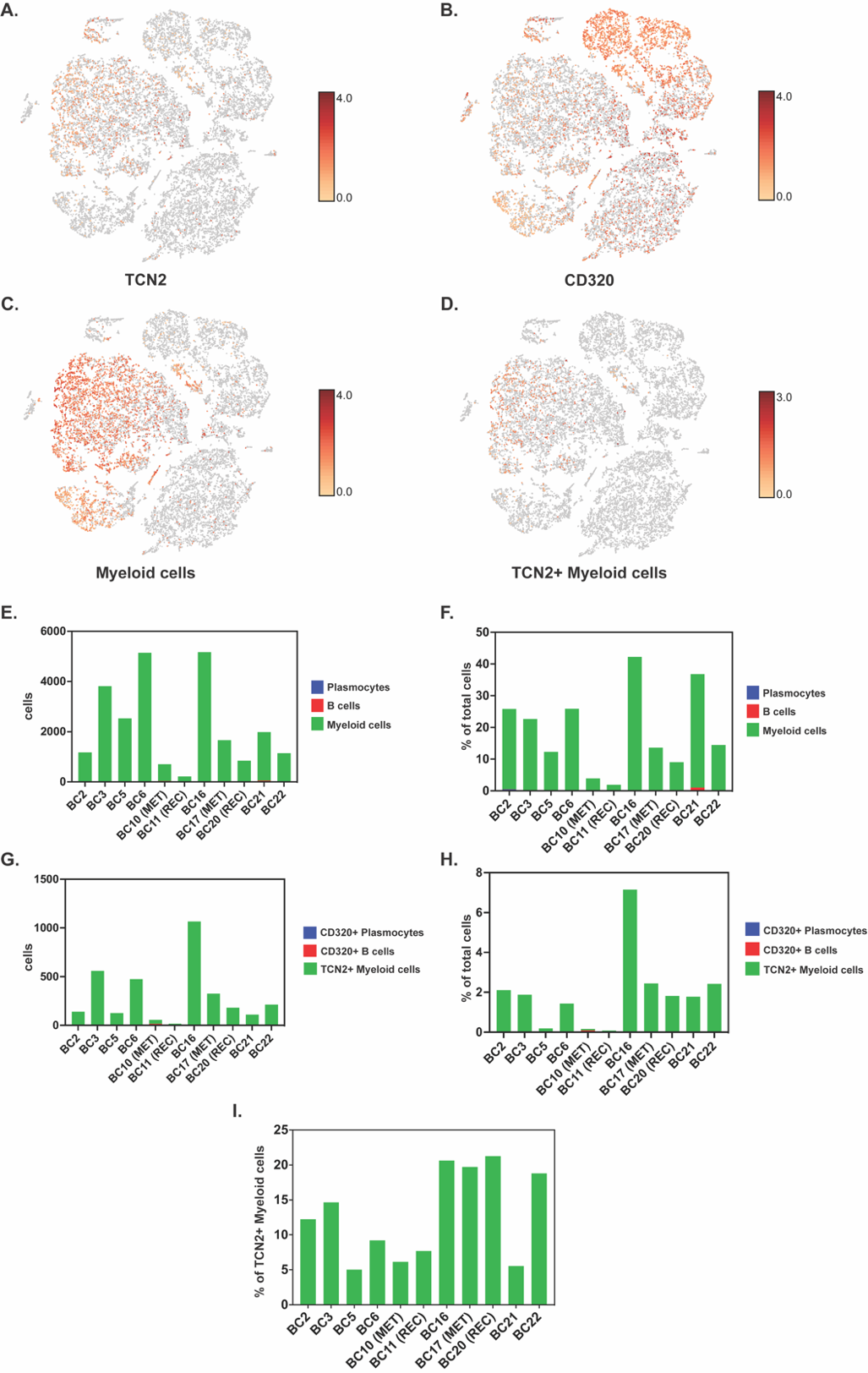
**

**Supplementary Figure 7 – scRNA-seq analysis of chemotherapy treated OSA tumors.** All clustering representative of patient BC3. (A) Representative log-normalized *TCN2* expression across various cellular clusters. (B) Representative log-normalized *CD320* expression across various cellular clusters. (C) Representative clustering of Myeloid cells with log-normalized expression of *CD68* and *LYZ* using the “Feature Min” function. (D) Representative clustering of *TCN2+* Myeloid cells with log-normalized expression of *TCN2, CD68*, and *LYZ* using the “Feature Min” function. (E) Quantification of total cells for the Plasmocytes, B cells, and Myeloid cells populations in all chemotherapy treated OSA tumors (n = 11). (F) Quantification of the percent (%) of total cells for Plasmocytes, B cells, and Myeloid cells population in all chemotherapy treated OSA tumors (n = 11). (G) Quantification of total cells for the *CD320+* Plasmocytes, *CD320+* B cells, and *TCN2+* Myeloid cells populations in all chemotherapy treated OSA tumors (n = 11). (H) Quantification of the percent (%) of total cells for *CD320+* Plasmocytes, *CD320+* B cells, and *TCN2+* Myeloid cells population in all chemotherapy treated OSA tumors (n = 11). (I) Quantification of the percent (%) of *TCN2+* Myeloid cells, relative to all Myeloid cells in all chemotherapy treated OSA tumors (n = 11).

**
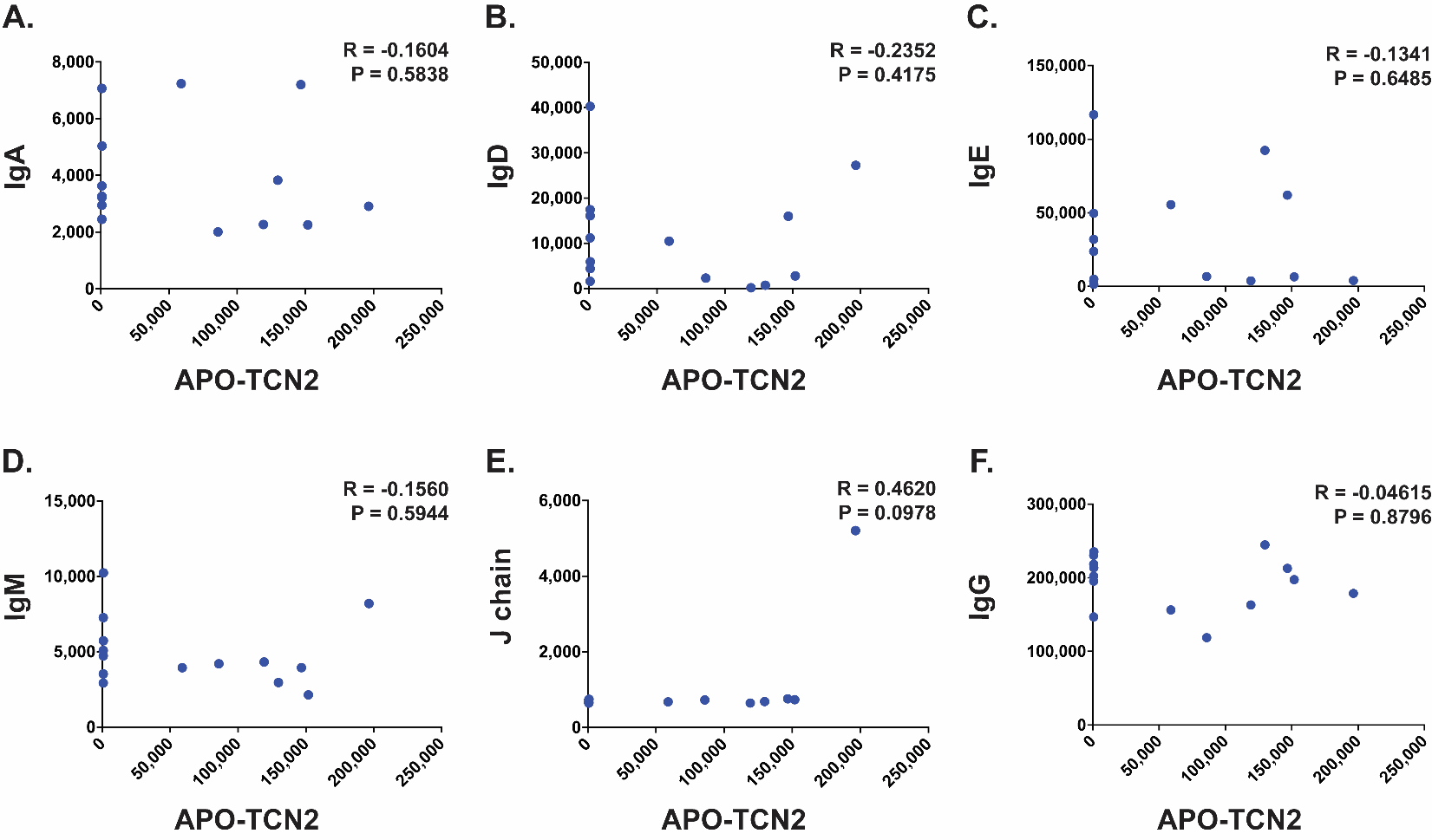
**

**Supplementary Figure 8 – Correlation analysis between APO-TCN2 and circulating immunoglobulins.** Spearman correlation analysis between measured APO-TCN2 (x-axis, RFU) and IgA [seq.11089.7] (A), IgD [seq.4916.2] (B), IgE [seq.4135.84] (C), IgM [seq.3069.52] (D), Immunoglobulin J chain [seq.15306.20] (E), IgG [seq.2744.57] (F) (y-axis, RFU) in naïve OSA patient plasma samples (n = 14). Spearman correlation coefficient (R) and associated P-value (P) presented on each scatter plot.

**
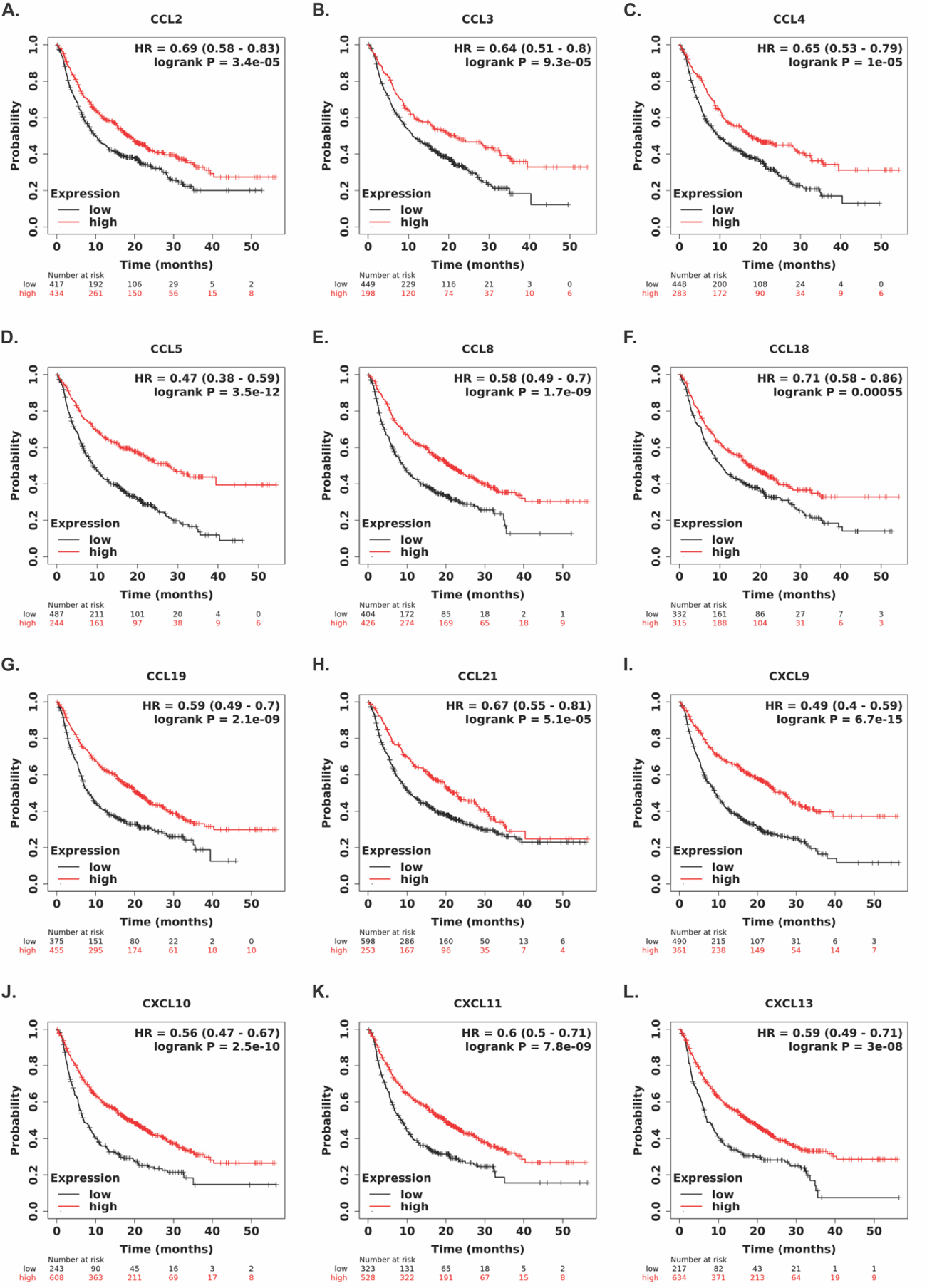
**

**Supplementary Figure 9 – KM plotter Immunotherapy survival analysis of the 12-CK TLS signature.** Overall survival analysis of *CCL2* (A)*, CCL3* (B)*, CCL4* (C)*, CCL5* (D)*, CCL8* (E)*, CCL18* (F)*, CCL19* (G)*, CCL21* (H)*, CXCL9* (I)*, CXCL10* (J)*, CXCL11* (K)*, CXCL13* (L). Each analysis included specimens from all solid tumors, collected pre-treatment, irrespective of immunotherapy target (anti-PD-1, anti-PD-L1, and anti-CTLA-4). Default KM Plotter settings were utilized with auto selection of best cutoff into high (red) and low (black) gene expression groups based on the calculation of all upper and lower quartiles with selection of the best performing threshold. KM survival curves with reported HR and log-rank P-value were constructed, with y-axis representing probability of survival and the x-axis representing time (months). The total number of patients at risk for each time point is reported.

**
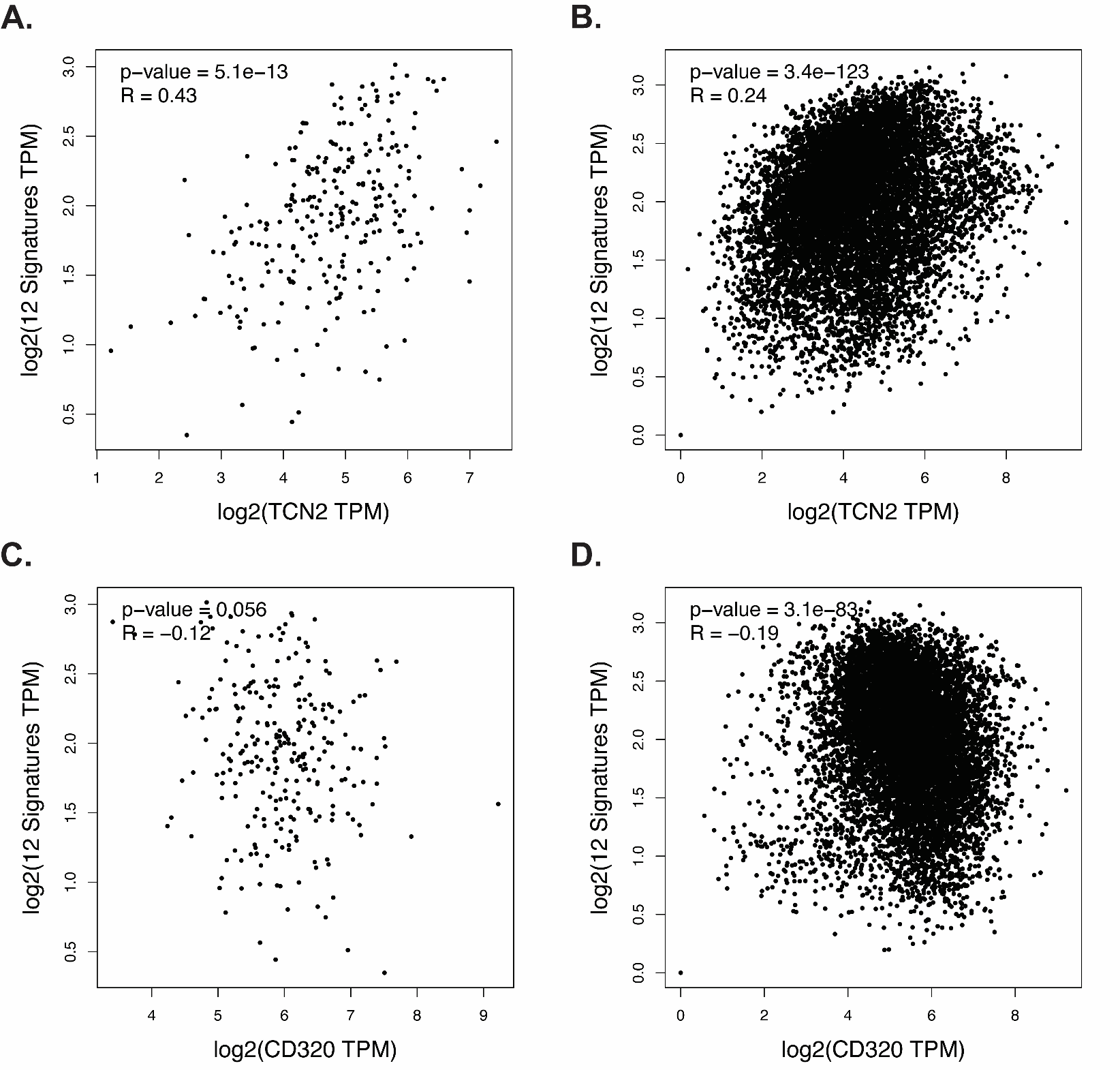
**

**Supplementary Figure 10 – TCGA correlation analysis of the 12-CK TLS signature using GEPIA2.** Multi-gene Spearman correlation analysis between *TCN2* and the 12-CK TLS signature using either only the TCGA-SARC gene expression dataset (A) or all tumor types of the TCGA were conducted (B). Multi-gene Spearman correlation analysis between *CD320* and the 12-CK TLS signature using either only the TCGA-SARC gene expression dataset (A) or all tumor types of the TCGA were conducted (B). For each correlation analysis, the log2(TPM) of the gene of interest (*TCN2* or *CD320*) was plotted on the x-axis while the log2(TPM) of the 12-CK gene signature is plotted on the y-axis. The resulting Spearman correlation coefficients (R) and P-values are reported.

| **Gene \| Gene** | **Mye_C7_C1QB \| Mye_C4_C1QA**  **(172.542)** | **Gene \| Gene** | **Mye_C3_LYZ \| Mye_C4_C1QA**  **(165.674)** | **Gene \| Gene** | **Mye_C6_S100A9 \| Mye_C4_C1QA**  **(157.193)** |
| --- | --- | --- | --- | --- | --- |
| CD74_MIF | 1.616 | CD74_MIF | 1.608 | CD74_MIF | 1.726 |
| HLA-DPB1_TNFSF13B | 1.421 | LGALS9_CD44 | 1.462 | CD74_COPA | 1.516 |
| LGALS9_CD44 | 1.419 | HLA-DPB1_TNFSF13B | 1.453 | TNFRSF1B_GRN | 1.502 |
| SPP1_CD44 | 1.414 | CD74_COPA | 1.398 | PLAUR_a4b1 complex | 1.442 |
| CD74_COPA | 1.406 | TNFRSF1A_GRN | 1.371 | HLA-F_LILRB2 | 1.438 |
| TNFRSF1B_GRN | 1.398 | TNFRSF1B_GRN | 1.318 | TNFRSF1A_GRN | 1.436 |
| TNFRSF1A_GRN | 1.357 | LGALS9_CD47 | 1.304 | C5AR1_RPS19 | 1.427 |
| C5AR1_RPS19 | 1.334 | PLAUR_a4b1 complex | 1.293 | CD44_HBEGF | 1.39 |
| LAIR1_LILRB4 | 1.317 | ANXA1_FPR3 | 1.293 | CD74_APP | 1.378 |
| HLA-F_LILRB2 | 1.29 | LGALS9_LRP1 | 1.274 | LGALS9_CD44 | 1.348 |
| CD99_PILRA | 1.286 | LGALS9_HAVCR2 | 1.27 | HLA-F_LILRB1 | 1.339 |
| CD74_APP | 1.268 | CD74_APP | 1.26 | HLA-DPB1_TNFSF13B | 1.332 |
| LGALS9_CD47 | 1.261 | HLA-F_LILRB2 | 1.255 | ANXA1_FPR3 | 1.323 |
| LGALS9_LRP1 | 1.231 | CD44_HBEGF | 1.226 | ANXA1_FPR1 | 1.239 |
| LGALS9_HAVCR2 | 1.226 | LAIR1_LILRB4 | 1.21 | LGALS9_CD47 | 1.189 |
| ANXA1_FPR3 | 1.201 | ANXA1_FPR1 | 1.21 | NAMPT_P2RY6 | 1.182 |
| HLA-F_LILRB1 | 1.191 | MIF_TNFRSF14 | 1.208 | CD99_PILRA | 1.164 |
| MIF_TNFRSF14 | 1.176 | CD99_PILRA | 1.189 | LGALS9_LRP1 | 1.16 |
| SPP1_a4b1 complex | 1.162 | C5AR1_RPS19 | 1.182 | LAIR1_LILRB4 | 1.155 |
| CD44_HBEGF | 1.135 | HLA-F_LILRB1 | 1.156 | LGALS9_HAVCR2 | 1.155 |

**Supplementary Table 1 – Top 3 interactions defined by cell-cell communication analysis for Mye_C4_C1QA of the SKCM1 dataset, with effect score presented in parenthesis.** Top 20 receptor-ligand interactions (gene | gene) and their gene expression driving the overall effect score between interacting cellular clusters.

| **Gene \| Gene** | **Mye_C4_C1QA \| B_C2_IGHG1**  **(124.479)** | **Gene \| Gene** | **Mye_C7_C1QB \| B_C2_IGHG1**  **(121.59)** | **Gene \| Gene** | **Mye_C3_LYZ \| B_C2_IGHG1**  **(120.367)** |
| --- | --- | --- | --- | --- | --- |
| CD74_MIF | 1.852 | CD74_MIF | 1.884 | CD74_MIF | 1.877 |
| C5AR1_RPS19 | 1.619 | C5AR1_RPS19 | 1.629 | CD74_COPA | 1.507 |
| CD74_COPA | 1.482 | CD74_COPA | 1.514 | C5AR1_RPS19 | 1.477 |
| LGALS9_CD44 | 1.307 | LGALS9_CD44 | 1.306 | LGALS9_CD44 | 1.35 |
| SPP1_CD44 | 1.194 | SPP1_CD44 | 1.301 | LGALS9_CD47 | 1.136 |
| TNFRSF1A_GRN | 1.13 | PLD2_ARF1 | 1.125 | MIF_TNFRSF14 | 1.127 |
| LGALS9_CD47 | 1.093 | TNFRSF1B_GRN | 1.12 | TNFRSF1A_GRN | 1.092 |
| TNFSF13_TNFRSF17 | 1.067 | MIF_TNFRSF14 | 1.095 | LGALS9_SLC1A5 | 1.058 |
| TNFRSF1B_GRN | 1.06 | LGALS9_CD47 | 1.093 | PLD2_ARF1 | 1.054 |
| MIF_TNFRSF14 | 1.059 | TNFRSF1A_GRN | 1.078 | TNFRSF1B_GRN | 1.04 |
| PLD2_ARF1 | 1.053 | TNFSF13_TNFRSF17 | 1.061 | HLA-C_FAM3C | 1.017 |
| LGALS9_SLC1A5 | 1.015 | HLA-C_FAM3C | 1.031 | TNFSF13_TNFRSF17 | 1.005 |
| HLA-C_FAM3C | 1.015 | LGALS9_SLC1A5 | 1.015 | CD74_APP | 0.983 |
| CD74_APP | 0.958 | CD74_APP | 0.991 | HLA-DPA1_TNFSF9 | 0.971 |
| TNFSF13_TNFRSF14 | 0.955 | CD94:NKG2A_HLA-E | 0.951 | PLAUR_a4b1 complex | 0.968 |
| HLA-DPA1_TNFSF9 | 0.938 | HLA-DPA1_TNFSF9 | 0.951 | HLA-DPB1_TNFSF13B | 0.897 |
| PLAUR_a4b1 complex | 0.93 | CD94:NKG2E_HLA-E | 0.95 | TNFSF13_TNFRSF14 | 0.893 |
| SIRPA_CD47 | 0.916 | TNFSF13_TNFRSF14 | 0.949 | HLA-DPB1_NRG1 | 0.875 |
| CCL3_IDE | 0.886 | EGFR_MIF | 0.921 | HLA-B_KIR3DL2 | 0.873 |
| GPR37L1_PSAP | 0.881 | HLA-E_KLRK1 | 0.889 | HLA-DRB1_OGN | 0.866 |

**Supplementary Table 2 – Top 3 interactions defined by cell-cell communication analysis for B_C2_IGHG1 of the SKCM1 dataset, with effect score presented in parenthesis.** Top 20 receptor-ligand interactions (gene | gene) and their gene expression driving the overall effect score between interacting cellular clusters.

|  | ***TCN2*** | ***CCL2*** | ***CCL3*** | ***CCL4*** |
| --- | --- | --- | --- | --- |
| ***TCN2*** | 1 |  |  |  |
| ***CCL2*** | 0.4544**** | 1 |  |  |
| ***CCL3*** | 0.407**** | 0.4587**** | 1 |  |
| ***CCL4*** | 0.3619**** | 0.4453**** | 0.8564**** | 1 |
|  | ***TCN2*** | ***CCL5*** | ***CCL8*** | ***CCL18*** |
| ***TCN2*** | 1 |  |  |  |
| ***CCL5*** | 0.4906**** | 1 |  |  |
| ***CCL8*** | 0.4933**** | 0.6532**** | 1 |  |
| ***CCL18*** | 0.4052**** | 0.5696**** | 0.5649**** | 1 |
|  | ***TCN2*** | ***CCL19*** | ***CCL21*** | ***CXCL9*** |
| ***TCN2*** | 1 |  |  |  |
| ***CCL19*** | 0.3837**** | 1 |  |  |
| ***CCL21*** | 0.2359**** | 0.7234**** | 1 |  |
| ***CXCL9*** | 0.3594**** | 0.5**** | 0.3598**** | 1 |
|  | ***TCN2*** | ***CXCL10*** | ***CXCL11*** | ***CXCL13*** |
| ***TCN2*** | 1 |  |  |  |
| ***CXCL10*** | 0.4344**** | 1 |  |  |
| ***CXCL11*** | 0.3866**** | 0.8785**** | 1 |  |
| ***CXCL13*** | 0.3491**** | 0.717**** | 0.6544**** | 1 |
|  | ***CD320*** | ***CCL2*** | ***CCL3*** | ***CCL4*** |
| ***CD320*** | 1 |  |  |  |
| ***CCL2*** | 0.2155**** | 1 |  |  |
| ***CCL3*** | 0.2006**** | 0.4587**** | 1 |  |
| ***CCL4*** | -0.0308 | 0.4453**** | 0.8564**** | 1 |
|  | ***CD320*** | ***CCL5*** | ***CCL8*** | ***CCL18*** |
| ***CD320*** | 1 |  |  |  |
| ***CCL5*** | 0.2489**** | 1 |  |  |
| ***CCL8*** | 0.4487**** | 0.6532**** | 1 |  |
| ***CCL18*** | 0.1884**** | 0.5696**** | 0.5649**** | 1 |
|  | ***CD320*** | ***CCL19*** | ***CCL21*** | ***CXCL9*** |
| ***CD320*** | 1 |  |  |  |
| ***CCL19*** | 0.215**** | 1 |  |  |
| ***CCL21*** | 0.1329*** | 0.7234**** | 1 |  |
| ***CXCL9*** | 0.1369*** | 0.5**** | 0.3598**** | 1 |
|  | ***CD320*** | ***CXCL10*** | ***CXCL11*** | ***CXCL13*** |
| ***CD320*** | 1 |  |  |  |
| ***CXCL10*** | 0.2315**** | 1 |  |  |
| ***CXCL11*** | 0.1962**** | 0.8785**** | 1 |  |
| ***CXCL13*** | 0.0728* | 0.717**** | 0.6544**** | 1 |

**Supplementary Table 3 – KM plotter Immunotherapy correlation analysis between *TCN2*, *CD320* and the chemokines of the 12-CK signature.** Spearman correlation analysis utilizing specimens from all solid tumors, collected pre-treatment (pretreatment acquisition), irrespective of immunotherapy target (anti-PD-1, anti-PD-L1, and anti-CTLA-4). Correlation coefficient and P-value are presented, with * = P <0.05, ** = P < 0.01, *** = P < 0.001, **** = P < 0.0001.
